# Polygenic burden has broader impact on health, cognition, and socioeconomic outcomes than most rare and high-risk copy number variants

**DOI:** 10.1038/s41380-021-01026-z

**Published:** 2021-02-01

**Authors:** Elmo Christian Saarentaus, Aki Samuli Havulinna, Nina Mars, Ari Ahola-Olli, Tuomo Tapio Johannes Kiiskinen, Juulia Partanen, Sanni Ruotsalainen, Mitja Kurki, Lea Martta Urpa, Lei Chen, Markus Perola, Veikko Salomaa, Juha Veijola, Minna Männikkö, Ira M. Hall, Olli Pietiläinen, Jaakko Kaprio, Samuli Ripatti, Mark Daly, Aarno Palotie

**Affiliations:** 1 Institute for Molecular Medicine Finland FIMM, University of Helsinki, Helsinki, Finland; 2 Finnish Institute for Health and Welfare, Helsinki, Finland; 3 Stanley Center for Psychiatric Research, The Broad Institute of Harvard and MIT, Cambridge, MA USA; 4 Analytical and Translational Genetics Unit, Massachusetts General Hospital, Boston, USA; 5 Department of Genetics, Yale School of Medicine, New Haven, CT USA; 6 McDonnell Genome Institute, Washington University School of Medicine, St. Louis, MO USA; 7 Research Unit of Clinical Neuroscience, University of Oulu & Oulu University Hospital, Oulu, Finland; 8 Northern Finland Birth Cohorts, Infrastructure for Population Studies, Faculty of Medicine, University of Oulu, Oulu, Finland; 9 Stem Cell and Regenerative Biology, Harvard University, Cambridge, USA; 10 Neuroscience Center, Helsinki Institute of Life Science, University of Helsinki, Helsinki, Finland; 11 Department of Public Health, University of Helsinki, Helsinki, Finland; 12 Analytic and Translational Genetics Unit, Department of Medicine, Department of Neurology and Department of Psychiatry, Massachusetts General Hospital, Boston, MA USA

**Keywords:** Genetics, Predictive markers, Schizophrenia, Bipolar disorder, Depression

## Abstract

Copy number variants (CNVs) are associated with syndromic and severe neurological and psychiatric disorders (SNPDs), such as intellectual disability, epilepsy, schizophrenia, and bipolar disorder. Although considered high-impact, CNVs are also observed in the general population. This presents a diagnostic challenge in evaluating their clinical significance. To estimate the phenotypic differences between CNV carriers and non-carriers regarding general health and well-being, we compared the impact of SNPD-associated CNVs on health, cognition, and socioeconomic phenotypes to the impact of three genome-wide polygenic risk score (PRS) in two Finnish cohorts (FINRISK, *n* = 23,053 and NFBC1966, *n* = 4895). The focus was on CNV carriers and PRS extremes who do not have an SNPD diagnosis. We identified high-risk CNVs (DECIPHER CNVs, risk gene deletions, or large [>1 Mb] CNVs) in 744 study participants (2.66%), 36 (4.8%) of whom had a diagnosed SNPD. In the remaining 708 unaffected carriers, we observed lower educational attainment (EA; OR = 0.77 [95% CI 0.66–0.89]) and lower household income (OR = 0.77 [0.66–0.89]). Income-associated CNVs also lowered household income (OR = 0.50 [0.38–0.66]), and CNVs with medical consequences lowered subjective health (OR = 0.48 [0.32–0.72]). The impact of PRSs was broader. At the lowest extreme of PRS for EA, we observed lower EA (OR = 0.31 [0.26–0.37]), lower-income (OR = 0.66 [0.57–0.77]), lower subjective health (OR = 0.72 [0.61–0.83]), and increased mortality (Cox’s HR = 1.55 [1.21–1.98]). PRS for intelligence had a similar impact, whereas PRS for schizophrenia did not affect these traits. We conclude that the majority of working-age individuals carrying high-risk CNVs without SNPD diagnosis have a modest impact on morbidity and mortality, as well as the limited impact on income and educational attainment, compared to individuals at the extreme end of common genetic variation. Our findings highlight that the contribution of traditional high-risk variants such as CNVs should be analyzed in a broader genetic context, rather than evaluated in isolation.

## Introduction

Large genomic rearrangements, called copy number variants (CNVs), have been identified as causative for a range of syndromes with neuropsychiatric traits [1–5]. While even most rare CNVs are considered non-deleterious, specific CNV types carry significant risk for severe neurodevelopmental and psychiatric disorders, and intellectual disability (ID) in particular [6, 7]. However, the penetrance and the contribution of CNVs to overall health is less studied. Kirov et al. [8] and others [3, 9, 10] showed that recurring CNVs associated with schizophrenia and ID-associated phenotypes have wide-ranging penetrance estimates. In two Finnish population-based studies, we have also shown that CNVs are associated with risk for schizophrenia, ID, lower educational attainment, and hearing impairment [11, 12].

Although the literature is still modest, previous work [3, 13–15] has suggested that CNVs can associate with lower general cognition and socioeconomic achievements in otherwise unaffected carriers. Kendall et al. [14] showed a cognitive and socioeconomic impact in unaffected carriers of rare disease-associated CNVs in the UK Biobank, and in a recent update [15] extended this analysis to reciprocal CNVs of the same regions. Crawford et al. [16] reported profound effects on non-cognitive traits, and health and mortality more generally, in CNV carriers in UK Biobank data. In neurodevelopmental disorders such as autism, de novo variant analysis [17] has shown that extending the phenotype from a dichotomous disease—no disease model into a spectrum of subclinical categories can yield a significant impact in otherwise unaffected carriers of risk variants. Männik et al. [13] showed that rare CNVs > 250 kb can be found in up to 10.5% of the population and correlate with ID and lower educational attainment. Non-neurological phenotypes such as anthropometric traits have also been shown [18] to associate with rare and recurring CNVs.

Polygenic risk scores (PRSs) have shown promise in investigating the complex genetic architecture of neuropsychiatric disorders. We [19] and others [20] have implicated the role of neuropsychiatric PRSs in ID and developmental delay. PRS for schizophrenia has been studied in the context of other neuropsychiatric traits [21], but earlier analyzes did not indicate a correlation between PRS for schizophrenia and mortality [22] or educational attainment [23] in individuals without schizophrenia. On the other hand, there is an established positive genetic correlation between educational attainment, intracranial volume, cognitive ability, schizophrenia, and bipolar disorder [24].

Both CNVs and high PRS are observed in the general population in individuals without obvious neurodevelopmental or neuropsychiatric disorders. Especially, given the expected high-risk nature of CNVs, the clinical evaluation and interpretation of their impact are challenging due to their relatively high frequency in unaffected individuals. So, if an adult with no history of severe neurological and psychiatric disorders (SNPDs) is observed to carry a disease-associated CNV, how much impact would that potentially have on the life trajectory?

We hypothesized that even if the majority of individuals carrying CNVs do not have a diagnosis of neurodevelopmental or neuropsychiatric diseases, CNVs might still contribute to the overall health and socioeconomic outcome. Thus, in participants without SNPD, we compared the impact of CNVs to the impact of the PRSs for educational attainment [24], schizophrenia [25], and general intelligence [26] on general health, morbidity, mortality, and socioeconomic burden. We analyzed these effects in two cohorts: one sampled at random from the Finnish working-age population (FINRISK), the other a Finnish birth cohort (Northern Finland Birth Cohort 1966; NFBC1966). Both cohorts link to national health records, enabling analysis of longitudinal health data and socioeconomic status data over several decades.

## Methods

We obtained phenotypic information on 35,231 individuals from the national FINRISK study [27], an on-going population study of the Finnish population. The data used for our study was received from the THL Biobank (study number: 39/2016). We selected a subset of 26,717 individuals based on the choice of SNP array applicable for CNV calling (Illumina HumanCoreExome). The NFBC1966 [28, 29] consisted of 5550 genotyped individuals (Illumina HumanCNV370 DNA beadchip). NFBC1966 participants were enrolled before birth and genotyped at age 31. After genotyping, we performed principal component (PC) analysis for FINRISK and NFBC1966. After excluding related individuals, duplicate samples, and PC outliers, 23,904 individuals in FINRISK and 4954 individuals in NFBC1966 remained for analysis.

We detected CNVs using a custom-built pipeline powered by PennCNV [30] and iPsychCNV [31] in both cohorts. Using our quality control criteria (Supplementary Materials), we removed 851 individuals from FINRISK and 59 individuals from NFBC1966. This resulted in a final count of 23,053 FINRISK and 4895 NFBC1966 participants. Table 1 presents the participant counts of both cohorts at the different QC steps.

**Table 1:** Study participants in different cohorts, and individuals remaining at each step.

Number of individuals	FINRISK	NFBC
**Phenotyped**	35,231	12,135
**Passing genotype QC**	23,053	4895
**With no SNPD**	22,210	4644

All individuals passing Sample QC were included in SNPD association analysis. Only individuals with no SNPD were included in the socioeconomic analysis.

CNV calls were included only if they had a minimum of ten consecutive probes supporting the call and were 100 kb or greater in length. We joined adjacent CNVs with similar copy number if the adjoining region was at most 20% of the full joined CNV. We identified as probable or potential artefacts any CNVs that overlapped an HLA- or immunoglobulin region by at least 50%, or that was within 500 kb of telomere or centromere region. Finally, we visualized all remaining CNV calls using the *visualize_cnv.pl* script distributed via the PennCNV package, and manually curated for obvious artefacts.

After filtering out samples and CNV calls of insufficient quality, we annotated CNVs as:

1. a *DECIPHER CNV* if at least 50% of the CNV overlapped a region associated with a CNV syndrome by the DECIPHER database [32];
2. an *ID gene deletion* if the CNV at least partially deleted 50% or more of the exons of a gene interpreted as monogenically causal for ID by the G2P gene set [33];
3. a *high pLI gene deletion* if the CNV deleted 50% or more of the exons of a gene with a high probability (≥0.95) of loss-of-function intolerance [34].

We denote as a “high-risk CNV” a CNV that matches any of these criteria or is greater than 1 Mb in size. Individuals carrying no high-risk CNV were used as controls (22,493 in FINRISK and 4724 in NFBC1966). We additionally tested CNVs specifically associated with the socioeconomic phenotypes in UK Biobank (educational attainment [15], household income [15], and medical consequences [16]) at a threshold of *p* < 0.001, to separately test for specific CNV impact (Supplementary Table 1).

We calculated PRS for educational attainment [24] (PRS_EA_), general intelligence [26] (PRS_IQ_), and schizophrenia [25] (PRS_SZ_) from previous large studies. LDpred was used to account for linkage disequilibrium among loci [35] using whole-genome sequencing data on 2690 Finns as the LD reference panel. Final scores were generated with PLINK2 [36, 37] by calculating the weighted sum of risk allele dosages for each single nucleotide polymorphism (SNP). We matched the case frequency for the total number of high-risk CNV carries (*n* = 573 in FINRISK, *n* = 171 in NFBC1966) by assigning case status to the same number of individuals at the extreme end of the respective distribution in each cohort. For PRS_EA_ and PRS_IQ_, we, therefore, analyzed the impact on the 744 individuals in the lowest extreme. For PRS_SZ_, we analyzed the 744 individuals in the highest extreme. We compared these PRS extremes to the middle 20–80% of the respective PRS distribution (13,831/23,053 in FINRISK, 2937/4895 in NFBC1966). This was done to prevent the overestimation of the impact of PRS outlier status that would result from comparing one outlier to its opposite extreme.

We performed a joint analysis to estimate the impact on income, education, and subjective health by grouping together individuals into three non-overlapping socioeconomic categories:

1. group, “low SES (socioeconomic status) and poor health”, consisted of participants with
  - Subjective health “average” (3) or worse AND.
  - Education level corresponding to lower secondary school or lower AND.
  - Household Income level 5/9 or lower.
2. group, “intermediate SES and health”, consisted of participants that
  - did NOT belong to group 1 AND.
  - did NOT belong to group 3.
3. group, “high SES and good health”, consisted of participants with
  - Subjective health “average” or better AND.
  - Education level corresponding to Upper Secondary School or higher AND.
  - Household Income level 5/9 or better.

The statistical models and phenotypic information are described in the Supplementary Methods and Supplementary Table 2.

## Results

**Fig. 1:**
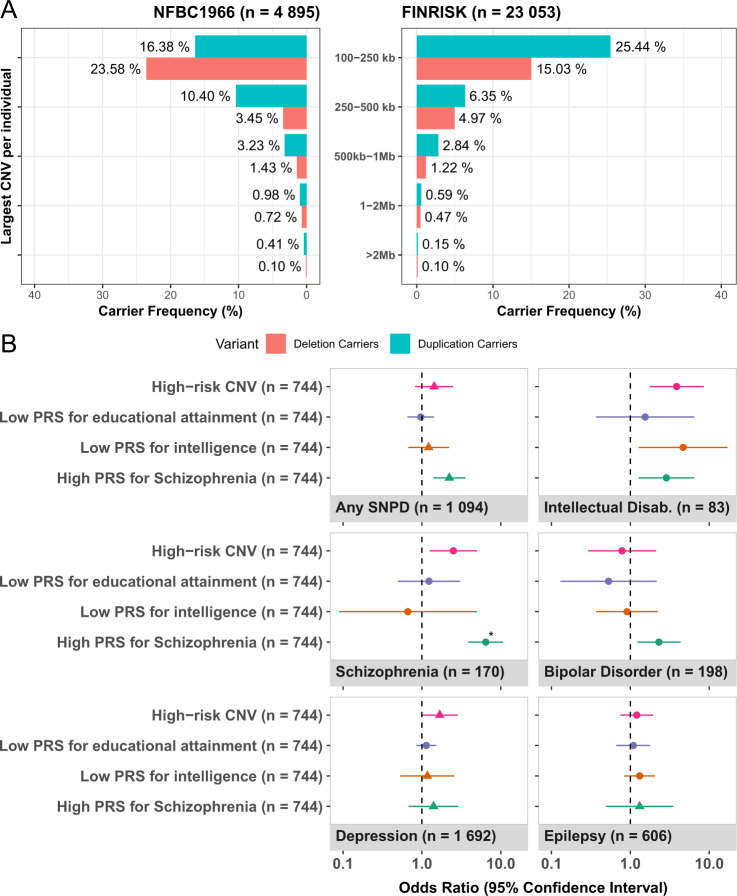
CNV size distribution and association with severe neurological and psychiatric disorders. **A** Size distribution of largest CNV/individual in the NFBC (left) and FINRISK (right) cohorts. **B** Meta-analysis of SNPD association in individuals with high-risk CNVs and matched extreme PRS outliers in the FINRISK and NFBC cohorts (*n* = 27,948). Overall, high-risk deletions except high pLI gene deletions were associated with SNPDs (Supplementary Fig. 3). We ascertained the disease associations using a logistic regression model, correcting for age (log), sex, PCs 1–10, and year of enrollment. ID was the most enriched phenotype in high-risk CNV carriers, though not significantly associated (OR = 3.9 [95% CI 1.7–8.6], *p*_adj_ = 0.080). In CNV subgroups (Supplementary Fig. 5), ID was associated with DECIPHER CNVs (OR = 11.8 [3.4–40.3], *p*_adj_ = 0.0074) and large deletions (OR = 9.9 [3.4–28.3], *p*_adj_ = 0.0018). CNV associations were overall stronger in NFBC (Supplementary Fig. 7) than FINRISK (Supplementary Fig. 6). Schizophrenia was reported more commonly among carriers of ID gene deletions (OR = 7.3 [1.7–31.0], *p* = 0.0070) and large deletions (OR = 4.9 [1.7–14], *p* = 0.0024) than among non-carriers, but these associations were not significant after correcting for multiple testing. If no considerable heterogeneity was observed (see Supplementary Methods), a fixed-effects model was assumed (circles), otherwise a random-effects model (triangles) was used in meta-analysis. The sole association significant after multiple testing (PRS_SZ_ and schizophrenia) is denoted with an asterisk.

To identify copy number variation, we ran PennCNV and iPsychCNV on genotype data from 23,053 FINRISK and 4895 NFBC1966 participants. This yielded 16,079 high-confidence calls (0.697 calls/individual) in FINRISK, and 3500 high-confidence calls in NFBC1966 (0.715 calls/individual), all larger than 100 kb. A deletion >100 kb was detected in 21.8% of FINRISK and 29.3% of NFBC1966 participants (Fig. 1A). A duplication >100 kb was detected in 35.4% of FINRISK and 31.4% of NFBC1966 participants. The size of most CNVs was no greater than 250 kb, criteria met by the largest variant of 69.0% of deletion carriers and 71.9% of duplication carriers in FINRISK, and 80.5% and 52.2% in NFBC1966, respectively. The overall distribution of the CNV sizes and types in NFBC1966 was similar to that of FINRISK, with frequencies in NFBC1966 being slightly higher in most size categories.

### Severe neurological and psychiatric disorders (SNPDs)

To confirm that CNV associations in FINRISK and NFBC1966 are in line with previous literature, we analyzed the associations of different CNV classes to SNPD traits (Supplementary Fig. 1). We selected these traits due to their established association with structural variants. The specific CNV classes referred to together as “high-risk CNVs”, consisted of calls that either: overlapped a previously reported region (“DECIPHER CNV” for CNVs overlapping at least 50% with DECIPHER regions; Supplementary Table 3); resulted in the loss of a high-impact gene (see “Methods”), or were large (>1 Mb). Table 2 presents frequencies of high-risk CNVs, along with the number of carriers affected by SNPD traits. The size distribution of CNVs was similar to previous studies [6, 13, 14] in both FINRISK and NFBC1966.

**Table 2:** Number and frequency of high-risk CNV carriers along with number and fraction of diagnosed SNPDs among carriers.

	Total		ID		SCZ		EPI		BD		MDD		Any SNPD^a^	
	*n*	%	*n*	%	*n*	%	*n*	%	*n*	%	*n*	%	*n*	%
FINRISK^b^	23,053	100%	55	0.24%	118	0.51%	501	2.17%	158	0.69%	1416	6.14%	843	3.66%
DECIPHER CNV	64	0.28%	1	1.56%	1	1.56%	1	1.56%	1	1.56%	7	10.94%	3	4.69%
ID gene deletion	43	0.19%	1	2.33%	1	2.33%	3	6.98%	1	2.33%	6	13.95%	5	11.63%
High pLI gene deletion	264	1.15%	0	0.00%	1	0.38%	5	1.89%	1	0.38%	15	5.68%	8	3.03%
>1 Mb deletion	129	0.56%	2	1.55%	2	1.55%	3	2.33%	1	0.78%	14	10.85%	7	5.43%
>1 Mb duplication	202	0.88%	2	0.99%	2	0.99%	5	2.48%	1	0.50%	17	8.42%	8	3.96%
Any high-risk CNV^c^	573	2.49%	4	0.70%	4	0.70%	13	2.27%	3	0.52%	46	8.03%	22	3.84%
NFBC1966^b^	4895	100%	28	0.57%	62	1.27%	105	2.15%	40	0.82%	276	5.64%	251	5.13%
DECIPHER CNV	37	0.76%	2	5.41%	2	5.41%	0	0.00%	0	0.00%	5	13.51%	5	13.51%
ID gene deletion	17	0.35%	0	0.00%	1	5.88%	1	5.88%	1	5.88%	3	17.65%	3	17.65%
High pLI gene deletion	78	1.55%	2	2.56%	3	3.85%	4	5.13%	1	1.28%	11	14.10%	9	11.54%
>1 Mb deletion	38	0.78%	2	5.26%	2	5.26%	0	0.00%	1	2.63%	6	15.79%	5	13.16%
>1 Mb duplication	53	1.08%	1	1.89%	0	0.00%	1	1.89%	0	0.00%	4	7.55%	3	5.66%
Any high-risk CNV^c^	171	3.49%	3	1.75%	5	2.92%	5	2.92%	1	0.58%	18	10.53%	14	8.19%

The first column shows the total number of participants in the cohort, along with the total number of carriers and their frequency. Consecutive columns indicate a number of carriers that have the relevant SNPD phenotype and the fraction of affected carriers. Not included are the diagnoses of childhood behavioral disorders and disorders of psychiatric development, due to the very low frequency of cases; they were however included in the “any SNPD” category. The percentage presented in the phenotype column is the fraction of carriers that have the disorder.

*ID* intellectual disability, *SCZ* schizophrenia, *EPI* epilepsy, *BD* bipolar disorder, *MDD* major depressive disorder.

^a^Depression was not included in this joint category.

^b^These rows indicate the total number of participants in the cohort and the total number of cases with an SNPD diagnosis.

^c^The number indicates individuals with any high-risk CNV. One individual might have more than one high-risk CNV, and one high-risk CNV can belong to several categories.

To enumerate the impact of high-risk CNVs compared to the common variant burden, we compared impacts to the frequency-matched extremes of the distribution of three PRS: educational attainment (PRS_EA_), intelligence (PRS_IQ_), and schizophrenia (PRS_SZ_). Among individuals at the extreme end of PRS_SZ_, association with schizophrenia was stronger (OR = 6.4 [3.9–10.7], *p*_adj_ = 5.8 × 10^−11^) than among high-risk CNV carriers (Fig. 1B). PRS_SZ_ also showed a trend with SNPDs in general (OR = 2.2 [1.3–3.6], *p*_adj_ = 0.088). Individuals at the low extreme of PRS_IQ_ were enriched for ID (OR = 4.7 [1.2–17.1], *p* = 0.020) more modestly than most high-risk CNV subgroups (Supplementary Fig. 2) and not significant after correction for multiple testing. In individuals with low PRS_EA_, we observed no enrichment of SNPD (Supplementary Figs. 3 and 4).

Despite the disease associations of high-risk CNV carriers, we observed that 708/744 (95.2%) of high-risk CNV carriers had no SNPD [551/573 (96.2%) in FINRISK; 157/171 (91.8%) in NFBC1966].

### Socioeconomic impact in individuals without a diagnosed SNPD

In FINRISK, there were 22,210 individuals (96.3%), and in NFBC1966 4644 individuals (94.9%), who had no diagnosed SNPD (not counting depression). We wanted specifically to analyze high-risk CNV carriers that had no record of SNPD to establish whether there was any impact on the general quality of life by analyzing overall health, education, and socioeconomic outcomes in these individuals (Supplementary Fig. 5).

**Fig. 2:**
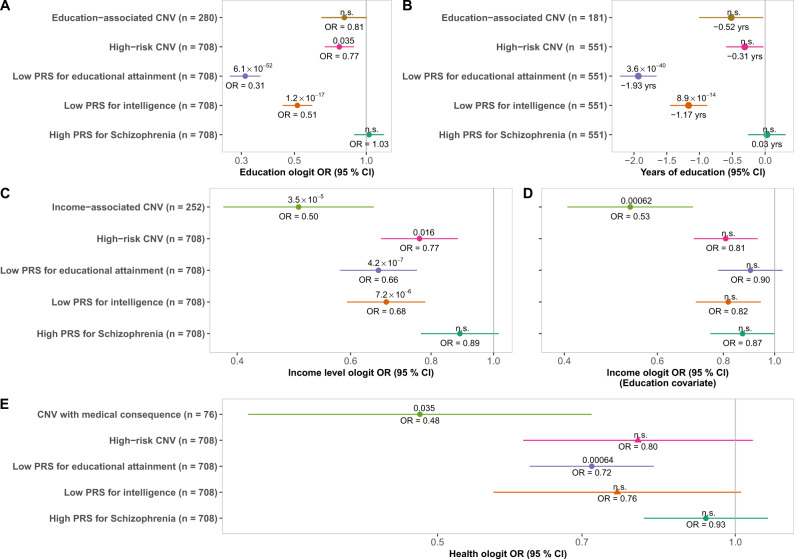
Socioeconomic impact of high-risk CNVs and PRSs in Finnish cohorts. **A** Ordered logit model of level of education for CNV types and PRS extremes in individuals with no SNPD (*n* = 25,944). Nine hundred and ten individuals were removed due to incomplete information on education, education reported as “ongoing”, or education reported as “other”. **B** Years of education lost due to CNV types and PRS extremes in FINRISK individuals with no SNPD (*n* = 21,961). Two hundred and forty-nine participants were removed from this analysis due to incomplete information on education. **C** Ordered logit model of household income (1–9) for CNV types and matched PRS extremes in individuals with no SNPD (*n* = 25,693). In total, 1161 participants were removed from analysis due to incomplete data on income. **D** When adjusting for education, most economic impacts from PRS and high-risk CNVs are accounted for. **E** Subjective health of CNV types and PRS extremes in individuals with no diagnosis of SNPD (*n* = 26,603). Subjective health was analyzed in an ordered logit model, where covariates were age, sex, and PCs 1–10. Two hundred and fifty-one participants were removed from the analysis due to incomplete data on subjective health. A circle denotes the use of a fixed-effect model; a triangle denotes a random effects model. Estimated effect is plotted with 95% confidence intervals, with point estimate denoted under the effect, and Bonferroni-corrected *p*-value denoted above.

PRS had a higher impact on education than high-risk CNVs (Fig. 2A). We modeled education in an ordered logistic regression (ologit) model for the level of education, correcting for age, sex, and PCs 1–10. In NFBC1966, we excluded individuals who reported their highest educational degrees as “unfinished” or “other” (final *n* = 3983). Our analysis indicated lower odds for the subsequent level of education among high-risk CNV carriers (OR = 0.77 [0.66–0.89]). Previously identified education-associated CNVs were not significantly associated with lower education (OR = 0.81 [0.64–1.01]). However, odds for subsequent level of education were even lower at the matched lowest extreme of PRS_EA_ (OR = 0.31 [0.26–0.37]) and PRS_IQ_ (OR = 0.51 [0.44–0.60]). The impact of high-risk CNVs was observed particularly with DECIPHER CNVs (OR = 0.51 [0.34–0.75]), large deletions (OR = 0.56 [0.41–0.76]), and high pLI gene deletions (OR = 0.72 [0.58–0.89]). The level of education was not significantly lower among individuals with a high PRS_SZ_.

We employed a linear regression model in FINRISK to estimate years of education (Fig. 2B), correcting for age, sex, year of baseline survey, and PCs 1–10. Individuals at the CNV-matched extreme of PRS_EA_ or PRS_IQ_ distributions had a stronger effect on education (*β*_EA_ = −19.3 [−2.22 to −1.65] and *β*_IQ_ = −1.17 [−1.46 to −0.88]) than high-risk CNVs (*β*_CNV_ = −0.31 [−0.60 to −0.02], *p*_adj_ = 1) or education-associated CNVs (*β*_EA-CNV_ = −0.52 [−1.01 to −0.02], *p*_adj_ = 1). On average, each additional +1 SD of PRS_EA_ added 0.84 years [0.79–0.89] of education, and each additional +1 SD of PRS_IQ_ 0.54 years [0.50–0.59] of education in FINRISK. Educational attainment in individuals at the high extreme of PRS_SZ_ was not lower than in controls (*β* = 0.03 [−0.25 to +0.32]).

Household income was conversely lower for carriers of income-associated CNVs than for individuals at the PRS extremes or for high-risk CNV carriers (Fig. 2C). We analyzed self-reported household income in an ologit model correcting for age, sex, PCs 1–10, and the number of individuals in the household. The model indicated a lower average income for individuals harboring income-associated CNVs (OR = 0.50 [0.38–0.66]) and high-risk CNVs (OR = 0.77 [0.66–0.89]) than non-carriers. Individuals at the lowest extreme of PRS_EA_ and PRS_IQ_ both reported lower household income (OR_EA_ = 0.66 [0.57–0.77]; OR_IQ_ = 0.68 [0.59–0.78]) than controls, though the confidence intervals overlapped with the OR of high-risk CNVs. Each additional +1 SD of PRS_EA_ in FINRISK increased household income (OR_EA_ = 1.22 [1.19–1.25]); the same was true for +1 SD of PRS_IQ_ in FINRISK (OR_IQ_ = 1.13 [1.10–1.16]). In high-risk CNV subgroups, household income was lower for carriers of large deletions (OR = 0.51 [0.38–0.70]) and high pLI gene deletions (OR = 0.66 [0.54–0.81]) (Supplementary Fig. 6). For the schizophrenia-based PRS, we did not see an effect on household income. When correcting for education (Fig. 2D), the impact of income-associated CNVs was largely unchanged (OR_adj_ = 0.53 [0.40–0.71]) whereas household income was not significantly lower in PRS extremes than in controls.

CNVs with reported medical consequences and PRS extremes were associated with lower subjective health, while high-risk CNVs reported similar health as controls (Fig. 2E). We analyzed subjective health in an ologit model corrected for age, sex, year of enrollment, and PCs 1–10. Carriers of CNVs with medical consequences consistently reported lower subjective health (OR = 0.48 [0.32–0.72]), while high-risk CNVs did not (OR = 0.80 [0.61–1.05]). The effect of high-risk CNVs differed between the cohorts (*I*^2^ = 59.9%), associating with lower health in NFBC1966 (Supplementary Fig. 7) but not in FINRISK (Supplementary Fig. 8). For common variation, individuals with the lowest PRS_EA_ had lower subjective health (OR_EA_ = 0.72 [0.61–0.83]). The impact of PRS_IQ_ (OR = 0.76 [0.56–1.02]) differed between the cohorts (*I*^2^ = 60.1%); low PRS_IQ_ was not associated with lower subjective health in NFBC1966 (*p* = 0.56) whereas it was in FINRISK (OR_IQ_ = 0.67 [0.57–0.80], *p*_adj_ = 1.5 × 10^−4^).

**Fig. 3:**
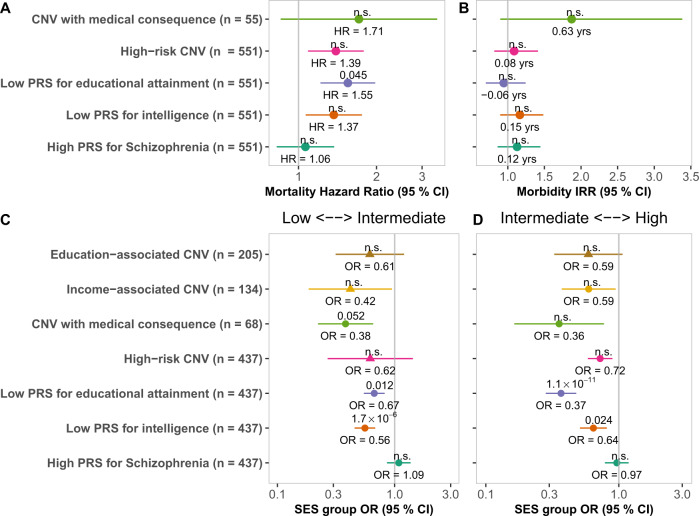
Health impact of high-risk CNVs and PRSs in Finnish cohorts. **A** Hazard ratios in a Cox regression model for mortality in unaffected carriers of high-risk CNVs and individuals at the PRS extremes in FINRISK (*n* = 22,210). ID gene deletions are not pictured as there were no deaths during follow-up for carriers of this type of CNV. **B** Incidence rate ratio (IRR) of high-risk CNVs and PRS extremes in a Poisson regression model of the Charlson comorbidity index in FINRISK individuals with no SNPD (*n* = 22,210). The incidence of one CCI unit was more than 3.5 higher in ID gene deletion carriers than in individuals with no high-risk CNV. **C**, **D** Impact of CNVs and PRS outlier status on socioeconomic status and health. The odds of low SES and poor health were highest for individuals with low PRS_IQ_, and to a lesser extent for individuals at the lowest extreme of PRS_EA_ (**A**). The odds of high SES and good health was lowest for individuals at the lowest extreme of PRS_EA_, and to a lesser extent for individuals at the lowest extreme of PRS_IQ_ (**B**). Effects meta-analyzed using a random-effects assumption are denoted by triangles, otherwise, a fixed-effect assumption was made. The Bonferroni-adjusted *p*-value is denoted above the point estimate of each variant.

Mortality was higher among individuals at the lowest PRS_EA_ extreme, but not in high-risk CNV carriers (Fig. 3A). Estimating mortality in FINRISK using a Cox regression model, we observed higher mortality among individuals at the lowest extreme of PRS_EA_ (HR = 1.55 [1.21–1.98]) compared to individuals within the middle 20–80% of the PRS_EA_ distribution. PRS_EA_ had no effect on mortality when taking lifestyle factors (smoking, BMI, alcohol consumption) into account. We did not observe higher mortality among individuals at the extremes of PRS_IQ_ (HR = 1.37 [1.06–1.76], *p*_adj_ = 1) or PRS_SZ_ (HR = 1.06 [0.82–1.37]), or among high-risk CNV carriers (HR = 1.39 [1.08–1.79], *p*_adj_ = 0.82) or CNVs with medical consequences (HR = 1.71 [0.85–3.43]). We did not estimate mortality in NFBC1966 due to the young age of the participants.

Only a small subgroup of high-risk CNVs showed higher general morbidity (Fig. 3B, Supplementary Fig. 9). To estimate morbidity, we computed the Charlson comorbidity index (CCI) for FINRISK individuals based on 20 phenotypes (Supplementary Table 4) with a high-impact on mortality. This data was not available for NFBC1966. We analyzed CCI in a Poisson regression model, correcting for age, sex, PCs 1–10 and year of enrollment. In FINRISK, the 36 ID gene deletion carriers had on average more than a three-fold incidence of CCI units (IRR = 3.4 [1.7–6.1], *p*_adj_ = 0.0097; Supplementary Fig. 9).

Analyzing the general quality of life, we found common variation, and PRS_EA_ in particular, to have a more substantial impact than high-risk CNVs (Fig. 3c, d). We estimated the general impact on both socioeconomic status and general well-being by grouping the cohort into three non-overlapping socioeconomic groups: low SES and poor health (group 1), intermediate SES and health (group 2), and high SES and good health (group 3; see Supplementary Methods and Supplementary Table 5). Students and participants aged > 65 were excluded (remaining *n* = 21,171). We tested variant impacts in a multinomial logistic regression model using group 2 as a reference, with sex, age, year of enrollment, and PCs 1–10 as covariates. We observed no group 1 or group 3 enrichment in individuals carrying high-risk CNVs (OR_intermediate↔high_ = 0.72 [0.58–0.90], *p*_adj_ = 0.28) nor in any CNV subgroup (Supplementary Fig. 10).

The effect of PRS was clearer. Individuals at the lowest extreme of PRS_EA_ and PRS_IQ_ were at higher odds of low SES and poor health (EA: OR_low↔intermediate_ = 0.67 [0.54–0.83]; IQ: OR_low↔intermediate_ = 0.56 [0.45–0.69]) and at lower odds of high SES and good health (EA: OR_intermediate↔high_ = 0.37 [0.28–0.48]; IQ: OR_intermediate↔high_ = 0.64 [0.50–0.82]). Individuals at the highest extreme of the PRS_SZ_ distribution did not show enrichment or depletion in any of the assigned groups. These analyzes suggest that the polygenic component is likely to have a higher predictive value for socioeconomic status and general well-being than the different CNV classes.

## Discussion

Here we find that the majority of working-age individuals in Finland carrying high-risk CNVs have a modest, if any, increased risk for major health or socioeconomic consequences. Only 4.8% of carriers had an associated neuropsychiatric disease, but this is not an accurate reflection of the true population frequency, as the most severe cases are underrepresented. We furthermore show no increase in overall mortality or morbidity in most high-risk CNVs. Unlike the relatively mild effect of most CNV classes, we observed a clear polygenic effect on socioeconomic outcome with educational attainment and IQ PRS scores. Belonging to the matched lowest PRS extremes (lowest 2.66%) of educational attainment or IQ had an overall stronger impact on the socioeconomic outcome than belonging to most high-risk CNV groups, and a generally stronger impact on health and survival, with the exception of household income-associated CNVs. These results imply that while on an individual level, high-risk variants can show a significant burden on specific neuropsychiatric disease risk and personal health, for carriers without such disease, the quality of life is expected to be comparable to that of the general population.

In general, the effect of deleterious rare variants (CNVs and protein truncating variants) on cognition and functional outcomes is well-established [3, 13, 15, 16, 38, 39]. Rare variants include both de novo variants and very rare variants that have arisen recently and have not yet been purged by negative selection [38]. Rare deleterious variants, including CNVs, can have a major impact on health outcomes for an individual and are thus under strong negative selection. However, such variants might not always have a strong phenotypic impact (incomplete penetrance), and as observed here, can have a very modest—if any—effect on well-being. The reason for this wide spectrum of outcomes remains speculative. From a genetic perspective, one hypothesis is that additional variants, both rare and common, modify the phenotypic outcome of a CNV carrier (Supplementary Figs. 11 and 12). This type of effect is observable in analyzes of hereditary breast and ovarian cancer in the UK Biobank [40] and in FinnGen [41], where strong-impacting variants’ penetrance is modified by compensatory polygenic effects. Another potential modifier could be a burden of rare variants, as reported by Ganna et al. [39], who observed a 2.9–3.1 month reduction in years of education for each disruptive or damaging mutation. There are fewer reports comparing the strength of associations of PRS and rare variants with each other. Both Kurki et al. [19] and Niemi et al. [20] demonstrated that both rare and common variants can contribute to ID, a heterogenic group of diseases that have typically been considered outcomes of high-impact deleterious variants. Other examples include cardiovascular diseases where the combination of rare and common variants has been studied [42, 43].

It is important to highlight that the rare deleterious CNVs studied here do not represent the full spectrum of the categories used. Many of the CNVs highlighted by the DECIPHER study [32] have only been reported in a handful of cases worldwide. Such high-penetrant variants and their associated syndromes are strongly selected against [44], as evidenced by the fact that DECIPHER CNVs were detected in 0.28% of FINRISK participants, while in the EGCUT cohort this same frequency was 0.71% [13]. That said, the aim of this study was to understand the socioeconomic and health outcomes of individuals without a clear SNPD diagnosis, so an underrepresentation of diagnostically severe cases is expected to increase the proportion of unaffected individuals among carriers.

The broad impact of CNVs, pleiotropic both clinically and subclinically, has been observed in numerous previous studies [13–16]. Case-control studies [6, 11] have used a number of CNV features for proxies of pathogenicity, and the ones used here are not exhaustive. Kendall et al. and Crawford et al., using the UK Biobank, have developed further CNV subclasses that better reflect socio-economic outcomes. Here we applied some of those new, proposed subclasses to see that e.g., CNVs associated to household income was replicated and exceeded the impact of PRSs. Subsequent studies will help to further clarify the characteristics of these potential new CNV subclasses.

The recent study by Männik et al. [13] in the Estonian population used a similar strategy as here to study the impact of CNVs on educational attainment by assigning CNVs to different classes, instead of focusing on specific chromosomal locations. They observed an association of deletions >250 kb to poorer school performance (ability to complete secondary education), which is in line with our previous study from Northern Finland [12], but neither of these previous studies reports on socioeconomic outcomes beyond education.

As stated above, the observed effect of polygenic scores was broader than that of structural variants. We observed strong effects in PRSs for intelligence and educational attainment on education, income and socioeconomic status. In line with previous studies, PRS for schizophrenia is mostly associated with schizophrenia with little effect on other traits, including subclinical or socioeconomic effect and educational attainment [22, 23]. Manifest schizophrenia both interrupts education and lowers SES, as these are associated with the chronic nature of the disease. The effect of PRSs on household income was modest after adjusting for education, but remained for income-associated CNVs. This effect of common variants is in line with previous epidemiological studies [45, 46], where education is a major factor influencing income. It also holds true in Nordic countries, where income levels are less unequal (as measured using the GINI index) than in many other countries [47].

The study design has some limitations. Firstly, as an observational and epidemiological study, no causal inference can be made. Second, analyzing CNVs in broad categories does not provide an insight into the effect of individual variants or loci, and can dilute the effect of these variants and loci. Third, with the chosen CNV calling method, we cannot separate between germline and somatic variants. Fourth, the calling accuracy of CNVs (Supplementary Methods) is outpaced by that of SNPs, potentially biasing the observed CNV impact. Fifth, as a large proportion of CNVs, are de novo, estimating their impact has somewhat different confounding factors than common variant-based polygenic scores. Sixth, diseases were mainly captured from hospital records without primary care data, potentially biasing the range of diseases captured. Finally, due to the underrepresentation of severe and highly penetrant CNVs, the observed frequency of 4.8% affected individuals does not accurately reflect the true disease risk [3, 6, 48]. A similar limitation was highlighted in the Kendall et al. study [14] in the UK Biobank.

There are also some differences between the two cohorts used. The 5-yearly collected data of the FINRISK cohort provides a good representation of the adult age spectrum from most geographical regions of Finland from different time points, different vocations, and different socioeconomic backgrounds. However, it also ensures that differences in the income and education distributions in the population are subject to long-term trends and fluctuations in the economical state and educational developments in the country [49–51]. While NFBC1966 is a birth cohort expected to give a more representative cross-section of the population, participants were sampled for DNA analysis at 31 years of age, selecting against early-onset severe cases. A third aspect is that the DNA chip used in NFBC1966 (CNV370) has 68.3% of the probe count of the CoreExome chip used in FINRISK CNV analysis. While this is not expected to impact noticeably on imputation and consequent PRS calculation, CNV resolution might be slightly different, despite the higher count of CNV calls in NBFC than in FINRISK.

The finding that the polygenic association with both education and the socioeconomic outcome is stronger than for most structural variants when cases with SNPD were excluded, highlights the polygenic background of these traits. The polygenic contribution in many complex traits has become evident in the wide GWAS literature. It seems that the genetic background of socioeconomic outcome has a strong polygenic component as well, which also modifies the effect of rare, stronger effect variants [40, 41].

We conclude that while contributing significantly to the risk for the development of neurological and psychiatric disorders, the majority of working-age individuals carrying high-risk CNVs have modest or no impact on morbidity and mortality, as well as limited impact on income and educational attainment. The vast majority of working-age individuals with observable high-risk CNVs have no associated SNPD. These unaffected carriers report on average lower subjective health, educational attainment, or income, but this impact is generally more modest than that observed among individuals at the extreme end of common genetic variation, highlighting the polygenic genetic background.

## Supplementary information

Supplemental Material Documentation

Supplementary Table 1: Counts and Frequencies of Phenotype-associated CNVs in FINRISK and NFBC

Supplementary Table 2: Disease endpoints considered

Supplementary Table 3: DECIPHER disease-associated CNV regions and frequencies

Supplementary Table 4: Charlson Comorbidity Phenotypes

Supplementary Table 5: Socioeconomic status groupings

Supplementary Figure 1: Correlation between neuropsychiatric disorders in FINRISK

Supplementary Figure 2: Meta-analysis of SNPD association with CNV subgroups

Supplementary Figure 3: SNPD association with CNV subgroups in FINRISK

Supplementary Figure 4: SNPD association with CNV subgroups in NFBC

Supplementary Figure 5: Spearman’s correlation between categorical socioeconomic endpoints in FINRISK

Supplementary Figure 6: Meta-analysis of level of household income in CNV subgroups

Supplementary Figure 7: Subjective health in different CNV subgroups in NFBC

Supplementary Figure 8: Subjective health in different CNV subgroups in FINRISK

Supplementary Figure 9: Charlson Comorbidity Index in different CNV subgroups in FINRISK

Supplementary Figure 10: Meta-analysis of impact of high-risk CNVs and PRS outlier status on socioeconomic grouping

Supplementary Figure 11: PRS_EA distribution in FINRISK CNV carriers vs non-carriers not affected by SNPD, by level of education

Supplementary Figure 12: PRS_EA distribution in FINRISK CNV carriers vs non-carriers not affected by SNPD, by years of education
