# Supplementary material for: Polygenic burden has broader impact on health, cognition, and socioeconomic outcomes than most rare and high-risk copy number variants
Source: Mol Psychiatry. 2021 Feb 1;26(9):4884–95. doi: 10.1038/s41380-021-01026-z (PMC8589645; doi:10.1038/s41380-021-01026-z)
Supplement: Supplementary file 1 — Supplemental Material Documentation [file 41380_2021_1026_MOESM1_ESM.docx]

# Supplementary Materials

## Study populations

### FINRISK population cohort

The National FINRISK Study, described in detail elsewhere[1], is an on-going population study of the Finnish population, where the population aged 25–74 years is sampled at random by groups of 200–250 from 10-year age groups and both sexes, such that 2 000 participants are recruited from each region. Independent samples of randomly chosen participants from the population are recruited every 5 years to join, gathering data through participant questionnaires, in-person clinical assessments, biosampling and accessing the Care Register for Health Care (HILMO), the Population Register Center (vital status), and Statistics Finland (cause of death). The total number of FINRISK participants included in this study was 35 231 individuals from the 1992, 1997, 2002, 2007 and 2012 surveys. We selected a subset of 26 717 individuals based on choice of SNP array applicable for CNV calling (Illumina HumanCoreExome).

Supplementary Table 2 presents the analyzed disease endpoints, together referred to as SNPDs. These diagnoses were ascertained in both cohorts by the diagnoses being either:

- present at Hospital Discharge or specialist outpatient visit in the HILMO register at least once,
- present as an underlying, immediate or contributing cause of death

When considering individuals with no SNPD, we did not exclude individuals with depression due to the high prevalence and potentially highly variable etiology of the disorder. Supplementary Figure 9 illustrates the correlation between the disease and socioeconomic endpoints.

### Northern Finland Birth Cohort (NFBC)

In order to readily approach the question of disease impact, we analyzed CNVs in the Northern Finland Birth Cohort 1966 (NFBC)[2]. Our genotyped sample consisted of 5 550 individuals who were recruited before birth and genotyped at age 31. NFBC is an unselected prospective birth cohort, consisting of all individuals born in the northernmost provinces of Finland in 1966. We have previously reported the CNV distribution in NFBC[3]. The 31-year field study included postal questionnaires and a clinical examination[4].

## Genotyping

We genotyped FINRISK individuals with Illumina HumanCoreExome DNA beadchip (542 585 probes, versions 12v1.0, 12v1.1 and 24v1.0). Imputation of the genotypes was done utilizing a Finnish population-specific reference panel of 3 775 high-coverage whole-genome sequences. We performed principal component (PC) analysis for 26 717 FINRISK individuals using 73 072 independent high-quality variants (autosomal single nucleotide polymorphisms with imputation info score > 0.99, 0.05 ≥ AF ≥ 0.95, HWE p-value > 0.001, call rate > 0.99, excluding high-LD regions, LD-pruned with r^2^ threshold 0.1 and 1 MB window-size). For PC analysis, we excluded 2 119 related individuals (kinship > 0.1). We calculated the first 10 PCs for 24 598 unrelated individuals, and extracted SNP weights. After excluding related individuals, duplicate samples and PC outliers, 23 904 individuals in FINRISK remained for CNV analysis.

For NFBC, all individuals in the analyzed sample were genotyped using Illumina HumanCNV370 DNA beadchip (370 405 probes, version 1). PC analysis was performed with the same approach as that of FINRISK. After excluding related individuals, duplicate samples and PC outliers, 4 954 individuals in NFBC remained for CNV analysis.

## CNV calling & analysis

CNVs were detected using a custom-built pipeline powered by PennCNV[5] and iPsychCNV[6], where samples were rejected if they had a large variance of intensity (Log R Ratio Standard Deviation > 0.3) or a large proportion of B allele frequency values outside of the typical range (B Allele Frequency drift > 0.005). Samples were further rejected if the analysis resulted in a total number of CNV calls (≥ 100 kb) more than four standard deviations above the mean (n(CNVs) ≥ 66). We removed 851 individuals (3.4 %) due to failing these criteria, bringing the total number of analyzed FINRISK individuals to 23 053.

We used the same pipeline to detect CNVs in NFBC as we did in the FINRISK population, with the default hidden Markov model matrix, and GC model chosen based on chip. Using the same quality control criteria as for FINRISK resulted in the removal of 59 individuals (1.2 %) from NFBC, with 4 895 individuals remaining for analysis.

CNV calls were included only if they had a minimum of 10 consecutive probes supporting the call and were 100 kb or greater in length. We joined adjacent CNVs with similar copy number if the adjoining region was at most 20 % of the full joined CNV. We identified as probable or potential artefacts any CNVs that overlapped a HLA- or immunoglobulin region by at least 50 %, or that was within 500 kb of telomere or centromere region. Finally, we visualized all remaining CNV calls using the *visualize_cnv.pl* script distributed via the PennCNV package, and manually curated for obvious artefacts.

To validate our pipeline, we also compared results with CNVs called using GenomeSTRIP on whole-genome sequencing data from a subset of 819 post-QC FINRISK individuals’ samples (unpublished data). This analysis showed that of our 565 high-confidence SNP-based calls (>100 kb), 534 (94.5 %) could be replicated in the WGS data set, suggesting a point estimate of 5.5 % for the false-positive rate. On the other hand, of the 5 693 WGS-based calls > 100 kb in this set, 321 (5.6 %) were detected using the SNP-based approach. Of these, 14 very common variants (6 deletions and 8 duplications) explain 95.0 % of the missed calls. The discrepancy between overlapping calls (534 vs 321) is caused by the used overlap threshold (>50%).

After filtering out samples and CNV calls of insufficient quality, we annotated CNVs as:

1. a **DECIPHER CNV** if at least 50 % of the CNV overlapped a region associated with a CNV syndrome by the DECIPHER database[7];
2. an **ID gene deletion** if the CNV at least partially deleted 50% or more of the exons of a gene interpreted as monogenically causal for intellectual disability by the G2P gene set[8];
3. a **high pLI gene deletion** if the CNV deleted 50% or more of the exons of a gene with a high probability (≥0.95) of loss-of-function intolerance[9].

We denote as a “high-risk CNV” any CNV that matches any of these criteria, or is greater than 1 Mb in size. In all statistical models, CNVs were analyzed in a case-control context, such that carriers were assigned as cases, and individuals carrying no high-risk CNV were assigned as controls (22 493 in FINRISK and 4 724 in NFBC1966). We additionally annotated CNVs specifically associated with the socioeconomic phenotypes (Educational attainment [10], Household Income [10] and Medical consequences [11]) to separately test for specific CNV impact.

## Polygenic Risk Scores (PRS)

We calculated PRS for educational attainment[12] (PRS_EA_), general intelligence[13] (PRS_IQ_), and schizophrenia[14] (PRS_SZ_). LDpred was used to account for linkage disequilibrium among loci[15] using whole-genome sequencing data on 2 690 Finns as the LD reference panel. The final scores were generated with PLINK2[16, 17] by calculating the weighted sum of risk allele dosages for each single nucleotide polymorphism (SNP). The weights were obtained from recent genome wide association studies (GWAS). The PRS for intelligence comprised 5 861 031 variants whereas PRS for educational attainment comprised 6 002 504 variants.

For analysis using PRS, we selected the most extreme values of the distribution, matching the frequency for the total number of high-risk CNV carries (n = 573 in FINRISK, n = 171 in NFBC). In other words, the most extreme 573/23 053 individuals (2.49 %) in FINRISK, and 171/4 895 individuals (3.49 %) in NFBC, altogether 744/27 948 individuals (2.66 %) of the respective PRS distributions, were chosen as cases. We chose to match the high-risk frequency to reflect the difference in frequency between the two cohorts. We considered the difference in frequency to reflect potential regional differences, and use of different genotype chips. For PRS_EA_ and PRS_IQ_, we analyzed impact on the 744 individuals in the lowest extreme. For PRS_SZ_, we analyzed the 744 individuals in the highest extreme. We recognized that comparing to the full rest of the PRS distribution would over-estimate the impact due to assigning individuals in the opposite end of the distribution as controls. We therefore compared these PRS extremes to the middle 20—80 % of the respective PRS distribution (13 831/23 053 in FINRISK, 2 937/4 895 in NFBC).

## Statistical analysis

Socioeconomic burden was estimated by analyzing impact on educational attainment, household income and general health in individuals with no diagnosis of SNPD (n = 26 854). Participants reported their educational attainment in the survey questionnaire both as years of education and level of education. The latter was set according to the following ordered category:

1. Basic education (ages 7—16 compulsory) or lower secondary education
2. Vocational education or equivalent
3. High school, upper secondary education or equivalent
4. Academic education (e.g. university), extended vocational education or equivalent

The participants estimated their gross household income as an ordered categorical variable (1—9) in the survey questionnaire. The categories were set according to 9-quantiles of the national reported household incomes of the previous year to the data collection, as made available by Statistics Finland (https://www.tilastokeskus.fi/index en.html).

General health was estimated by observing subjective health, the Charlson Comorbidity Index (CCI)[18], and risk of death during the follow-up period. The participants reported their subjective health as a response to the question, ”How do you find your health status?” on a scale of 1—5, representing ”Very good”, ”Quite good”, ”Average”, ”Quite bad”, and ”Very bad”, respectively. For analysis, we reversed the enumeration so that a higher score would represent a better subjective health.

We calculated CCI based on a set of 20 phenotypes (Supplementary Table 4) that we deemed to affect the short-term mortality of the individual. CCI was analyzed in a Poisson regression model, using sex, age (log), PCs 1—10, and year of participation (1992/1997/2002/2007/2012) as covariates. The lifestyle factors of current smoking status, weekly alcohol consumption (log transformation of alcohol consumption + 1), and BMI (log) were included as covariates during sensitivity analysis.

We used Cox’ proportional hazard model to estimate survival curves, hazard ratios (HRs) and 95 % confidence interval (95 % CI) in the survival analyses where age was used as the time scales. Follow-up started at the time of survey and ended at death or at the end of follow-up (December 31, 2014), whichever came first. In FINRISK the mean follow-up time was 13.31 years (Median 12.88 years, IQR [7.84-17.90] years) and in NFBC66 both genotype and phenotype information was collected at 31 years of age. We used R’s cox.zph function to test that the proportional assumption criteria applied in our survival models. Survival models were adjusted for sex, PCs 1—10, and year of enrollment. Sensitivity analysis for Cox regression included taking into account lifestyle factors (smoking, BMI, alcohol consumption), years of education, and vocation. Participants reported their vocation as one of seven categories in FINRISK.

We performed a joint analysis to estimate the impact on income, education, and subjective health. We grouped together individuals into three non-overlapping socioeconomic categories according to the following:

1. group, “low SES (Socioeconomic Status) and poor health”, consisted of participants with
   1. Subjective health “Average” (3) or worse
      AND
   2. Education level corresponding to Lower Secondary School or lower
      AND
   3. Household Income level 5/9 or lower
2. group, “intermediate SES and health”, consisted of participants that
   1. did NOT belong to group 1
      AND
   2. did NOT belong to group 3
3. group, “high SES and good health”, consisted of participants with
   1. Subjective health “Average” or better
      AND
   2. Education level corresponding to Upper Secondary School or higher
      AND
   3. Household Income level 5/9 or better

In FINRISK, Group 1 (low SES) constituted 20.8 %, Group 2 (intermediate SES) constituted 51.5 %, and Group 3 (high SES) constituted 27.7 % of the study sample. In NFBC1966, where we replicated this analysis, low SES was assigned to 12.8 %, intermediate SES to 52.7 %, and high SES to 34.5 % of the study sample. All three socioeconomic measures correlated considerably (Supplementary Figure 10), albeit at most 32 % (Spearman’s correlation). This provides statistical evidence that any one of the three measures provides substantial additional information on socioeconomic status in comparison to the other two measures.

To analyze the impact of variants on the odds of belonging to one of our assigned groups, we used a multinomial logistic regression model of the socioeconomic groups, using sex, age, year of enrollment and PCs 1—10 as covariates. We used the *multinom* function from the R package *nnet* to employ the multinomial model.

We performed both disease-enrichment and socioeconomic analyses in both FINRISK and NFBC whenever possible. The results were then meta-analyzed[19] using the inverse variance method. We used a fixed effect model in the meta-analysis by default. If heterogeneity was observed between the cohorts, defined as $I^{2}>50 \%$ or $p_{Q}<0.1$, then a random effects model was employed (using DerSimonian-Laird estimator for $\tau^{2}$)[20]. We corrected all *p*-values for multiple testing according to 8 variants (5 categories of CNV and 3 PRSs) being tested on 12 endpoints assumed to be independent (96 tests), in addition to 13 tests using 3 phenotype-associated CNVs – 109 independent tests in total. In figures, all *p*-values are Bonferroni-corrected; in the text, $p_{adj}$denote corrected *p*-values.

As part of our sensitivity analysis, we tested joint models where the impact of a high-risk variant with one or more PRS as covariates. We repeated this analysis with the addition of an interaction term between PRS and high-risk variant. Adding a PRS as covariate showed similar impact for both the tested high-risk CNV and the PRS in question, and no interaction term showed significant impact for any outcome after correction for multiple testing. We find that this effect is in line with the previously reported additive model for the impact of polygenic and rare variant burden[21].

## References

1. Borodulin K, Vartiainen E, Peltonen M, Jousilahti P, Juolevi A, Laatikainen T *et al.* Forty-year trends in cardiovascular risk factors in Finland. *European Journal of Public Health* 2015; **25**(3)**:** 539-546.

2. Rantakallio P. The longitudinal study of the Northern Finland birth cohort of 1966. *Paediatric and Perinatal Epidemiology* 1988; **2**(1)**:** 59-88.

3. Pietiläinen OP, Rehnström K, Jakkula E, Service SK, Congdon E, Tilgmann C *et al.* Phenotype mining in CNV carriers from a population cohort. *Human molecular genetics* 2011; **20**(13)**:** 2686-2695.

4. Sovio U, King V, Miettunen J, Ek E, Laitinen J, Joukamaa M *et al.* Cloninger's Temperament dimensions, socio-economic and lifestyle factors and metabolic syndrome markers at age 31 years in the Northern Finland Birth Cohort 1966. *Journal of health psychology* 2007; **12**(2)**:** 371-382.

5. Wang K, Li M, Hadley D, Liu R, Glessner J, Grant SF *et al.* PennCNV: an integrated hidden Markov model designed for high-resolution copy number variation detection in whole-genome SNP genotyping data. *Genome research* 2007; **17**(11)**:** 1665-1674.

6. Marcelo Bertalan h, idaElken. iPsychCNV v1.0 (Version v1.0). *Zenodo* 2016.

7. Firth HV, Richards SM, Bevan AP, Clayton S, Corpas M, Rajan D *et al.* DECIPHER: Database of Chromosomal Imbalance and Phenotype in Humans Using Ensembl Resources. *The American Journal of Human Genetics* 2009; **84**(4)**:** 524-533.

8. Wright CF, Fitzgerald TW, Jones WD, Clayton S, McRae JF, van Kogelenberg M *et al.* Genetic diagnosis of developmental disorders in the DDD study: a scalable analysis of genome-wide research data. *The Lancet* 2015; **385**(9975)**:** 1305-1314.

9. Lek M, Karczewski KJ, Minikel EV, Samocha KE, Banks E, Fennell T *et al.* Analysis of protein-coding genetic variation in 60,706 humans. *Nature* 2016; **536:** 285.

10. Kendall KM, Bracher-Smith M, Fitzpatrick H, Lynham A, Rees E, Escott-Price V *et al.* Cognitive performance and functional outcomes of carriers of pathogenic copy number variants: analysis of the UK Biobank. *The British journal of psychiatry : the journal of mental science* 2019**:** 1-8.

11. Crawford K, Bracher-Smith M, Owen D, Kendall KM, Rees E, Pardiñas AF *et al.* Medical consequences of pathogenic CNVs in adults: analysis of the UK Biobank. *Journal of Medical Genetics* 2019; **56**(3)**:** 131.

12. Okbay A, Beauchamp JP, Fontana MA, Lee JJ, Pers TH, Rietveld CA *et al.* Genome-wide association study identifies 74 loci associated with educational attainment. *Nature* 2016; **533**(7604)**:** 539-542.

13. Savage JE, Jansen PR, Stringer S, Watanabe K, Bryois J, de Leeuw CA *et al.* Genome-wide association meta-analysis in 269,867 individuals identifies new genetic and functional links to intelligence. *Nature Genetics* 2018; **50**(7)**:** 912-919.

14. Ripke S, Neale BM, Corvin A, Walters JTR, Farh K-H, Holmans PA *et al.* Biological insights from 108 schizophrenia-associated genetic loci. *Nature* 2014; **511**(7510)**:** 421-427.

15. Vilhjalmsson BJ, Yang J, Finucane HK, Gusev A, Lindstrom S, Ripke S *et al.* Modeling Linkage Disequilibrium Increases Accuracy of Polygenic Risk Scores. *Am J Hum Genet* 2015; **97**(4)**:** 576-592.

16. Shaun Purcell CC. PLINK version 2.0. vol. PLINK v2.00a2LM 64-bit Intel (9 Oct 2019).

17. Chang CC, Chow CC, Tellier LC, Vattikuti S, Purcell SM, Lee JJ. Second-generation PLINK: rising to the challenge of larger and richer datasets. *GigaScience* 2015; **4:** 7.

18. Charlson ME, Pompei P, Ales KL, MacKenzie CR. A new method of classifying prognostic comorbidity in longitudinal studies: development and validation. *Journal of chronic diseases* 1987; **40**(5)**:** 373-383.

19. Veroniki AA, Jackson D, Viechtbauer W, Bender R, Bowden J, Knapp G *et al.* Methods to estimate the between-study variance and its uncertainty in meta-analysis. 2016; **7**(1)**:** 55-79.

20. DerSimonian R, Laird N. Meta-analysis in clinical trials. *Controlled Clinical Trials* 1986; **7**(3)**:** 177-188.

21. Weiner DJ, Wigdor EM, Ripke S, Walters RK, Kosmicki JA, Grove J *et al.* Polygenic transmission disequilibrium confirms that common and rare variation act additively to create risk for autism spectrum disorders. *Nat Genet* 2017; **49**(7)**:** 978-985.

# Supplementary Table and Figure legends

**Supplementary Table 2: Disease endpoints considered.** ICD codes used in compilation of disease endpoints in FINRISK. All decimals more accurate than the prescribed code were included in the parent category. The total number of individuals with disease endpoints are presented in Table 2 of the manuscript.

**Supplementary Table 3: DECIPHER disease-associated CNV regions and frequencies.** Disease-associated CNVs as listed at the DECIPHER database website, annotated by type, location, size and frequency. Only CNVs >100 kb were used in this study.

**Supplementary Table 4: Charlson Comorbidity Phenotypes.** Phenotype selections used in compilation of Charlson comorbidity index (CCI). CCI was calculated for each listed phenotype, corresponding to the appearance of a comorbidity belonging to that category during follow-up time. Total individual CCI was determined as the sum of all phenotype CCIs without discrimination or ranking.

**Supplementary Table 5: Socioeconomic status groupings.** Descriptive statistics for the socioeconomic groups used in the multinomial regression model. Group 1 (low SES and poor health) consisted of individuals with low income, at most lower secondary education, and at most ”average” subjective health. Group 3 (high SES and good health) consisted of individuals with high income, at least ”average” subjective health, and at least upper secondary education. Group 2 (Intermediate SES and health) consisted of individuals not assigned to Group 1 or Group 3. Individuals assigned to low SES and poor health were on average more likely to smoke, had a smaller female fraction, and a higher death rate (in FINRISK) than individuals assigned to intermediate or high SES. Alcohol consumption differed in the two cohorts, in that high SES reported higher median drinking than low SES in FINRISK, whereas in NFBC the opposite trend was observed. Disability pension was seen at a far lower rate in NFBC due to the young and unvaried age (all recruited in the same year at age 31), whereas FINRISK participants are recruited from a far wider age range (25—75). The socioeconomic grouping does not include individuals with an SNPD diagnosis, students, or individuals of age ≥65 years (FINRISK: n = 17,576; NFBC: n = 3,595).

**Supplementary Figure 1: Meta-analysis of SNPD association with CNV subgroups.** Most high-risk CNVs showed a higher association with ID than the extreme end of PRS for intelligence did. Specific CNV subgroups reached similar or higher level of association with most neurological disorders, and ID gene deletions in particular. Point estimates for odds ratios are denoted with a circle if a fixed-effects model was used, and with a triangle if a random-effects model was used.

**Supplementary Figure 2: SNPD association with CNV subgroups in FINRISK.** Most high-risk CNVs showed a higher association with ID than PRS extremes did. Specific CNV subgroups reached similar or higher level of association with most neurological disorders, and ID gene deletions in particular. Schizophrenia was not associated with any high-risk CNV subgroup. Point estimates for odds ratios are denoted with a circle (triangle if significant after multiple testing correction. Unadjusted p-values are denoted on the left-hand side.

**Supplementary Figure 3: SNPD association with CNV subgroups in NFBC.** Most high-risk CNVs showed a higher association with ID than PRS extremes did. Specific CNV subgroups reached similar or higher level of association with most neurological disorders, including schizophrenia. Point estimates for odds ratios are denoted with a circle (triangle if significant after multiple testing correction). Unadjusted p-values are denoted on the left-hand side.

**Supplementary Figure 4: Meta-analysis of level of household income in CNV subgroups.** We modelled self-reported Household Income (1—9) in an ordered logistic regression model (left), meta-analyzed from both NFBC and FINRISK using a fixed effect assumption. Model uses Sex, log(Age), Number of Individuals living in the same household, and PCs 1—10 as covariates. When adjusting for education (right), a significant portion of impact from PRS is accounted for, while most high-risk CNVs have similar impact. Denoted above point estimates are Bonferroni-corrected p-values.

**Supplementary Figure 5: Subjective health in different CNV subgroups in NFBC.** Only ID gene deletions associated with lower subjective health in an ordered logistic regression model, while both low PRS for educational attainment and low PRS for intelligence did. The impact of ID gene deletions was stronger than the impact of belonging to the low PRS extreme of either score. Point estimates for odds ratios are denoted by a circle (triangle if significant after multiple testing correction), with enumerated point estimate below and unadjusted p-value above.

**Supplementary Figure 6: Subjective health in different CNV subgroups in FINRISK.** Only ID gene deletions associated with lower subjective health in an ordered logistic regression model, while both low PRS for educational attainment and low PRS for intelligence did. The impact of ID gene deletions was stronger than the impact of belonging to the low PRS extreme of either score. Point estimates for odds ratios are denoted by a circle (triangle if significant after multiple testing correction), with enumerated point estimate below and unadjusted p-value above.

**Supplementary Figure 7: Charlson Comorbidity Index in different CNV subgroups in FINRISK.** ID gene deletions had CCI points at over three-fold the rate of controls, while no other CNV subgroup or PRS extreme showed increased rates of CCI. Point estimates for incidence rate ratios are denoted by a circle, with enumerated point estimate below and adjusted p value above.

**Supplementary Figure 8: Meta-analysis of impact of high-risk CNVs and PRS outlier status on socioeconomic grouping.** The odds of having a general impact on socioeconomic resources and health (group 1) was higher for DECIPHER CNVs (left) than PRSs. The odds of escaping impact altogether (high SES) was lower for individuals at the lowest extreme of PRS_EA_, and carriers of DECIPHER CNVs (right). A circle denotes the use of a fixed-effect model, a triangle denotes a random-effects model. Random effects were assumed when the effect was substantially heterogeneous between the different cohorts. Specific values of point estimates are denoted below the odds ratio, with Bonferroni-corrected p-values above.

**Supplementary Figure 9: Correlation between neuropsychiatric disorders in FINRISK.** Most neurological and psychiatric disorders showed little or no correlation with each other. Notable correlation was observed between schizophrenia and bipolar disorder, and between bipolar disorder and depression.

**Supplementary Figure 10: Spearman’s correlation between categorical socioeconomic endpoints in FINRISK.** A positive correlation between all the categorical variables of level of education, subjective health and household income was observed.

**Supplementary Figure 11: PRS_EA_ distribution in FINRISK CNV carriers vs non-carriers not affected by SNPD, by level of education.** PRS_EA_ distribution in CNV carriers without SNPD is similar to that of education-level matched non-carriers without SNPD.

**Supplementary Figure 12: PRS_EA_ distribution in FINRISK CNV carriers vs non-carriers not affected by SNPD, by years of education.** Years of education over PRS_EA_ distribution in FINRISK CNV carriers without SNPD shows a similar positive correlation (R^2^_adj_ = 0.04) to the positive correlation between PRS_EA_ and years of education observed in FINRISK non-carriers without SNPD (R^2^_adj_ = 0.049).
