# Supplementary material for: Polygenic burden has broader impact on health, cognition, and socioeconomic outcomes than most rare and high-risk copy number variants
Source: Mol Psychiatry. 2021 Feb 1;26(9):4884–95. doi: 10.1038/s41380-021-01026-z (PMC8589645; doi:10.1038/s41380-021-01026-z)
Supplement: Supplementary file 2 — Supplementary Table 1: Counts and Frequencies of Phenotype-associated CNVs in FINRISK and NFBC [file 41380_2021_1026_MOESM2_ESM.pdf]

CNV	Educational attainment	Household Income	Medical consequences	Count (FINRISK)	Frequency (FINRISK)	Count (NFBC1966)	Frequency (NFBC1966)
TAR dup	x	x	x	5	0.02 %	0	0.00 %
1q21.1 del		x	x	8	0.03 %	2	0.04 %
1q21.1 dup	x	x	x	4	0.02 %	2	0.04 %
NRXN1 del		x	x	2	0.01 %	2	0.04 %
2q13 del		x		2	0.01 %	0	0.00 %
3q29 del	x	x	x	0	0.00 %	0	0.00 %
3q29 dup			x	0	0.00 %	0	0.00 %
WBS dup	x	x	x	0	0.00 %	0	0.00 %
10q11.21q11.23 del	x	x		1	0.00 %	0	0.00 %
15q11.2 del	x	x		66	0.29 %	13	0.27 %
15q11.2 dup	x			82	0.36 %	18	0.37 %
15q11q13 dup (BP3-BP4)		x	x	0	0.00 %	0	0.00 %
15q11 dup (PW/AS)	x	x	x	1	0.00 %	0	0.00 %
15q13.3 del			x	5	0.02 %	1	0.02 %
15q13.3 dup	x	x		1	0.00 %	4	0.08 %
16p13.11 del	x	x		7	0.03 %	1	0.02 %
16p13.11 dup	x	x	x	9	0.04 %	7	0.14 %
16p12.1 del	x	x	x	1	0.00 %	6	0.12 %
16p11.2 distal del	x	x	x	0	0.00 %	1	0.02 %
16p11.2 distal dup	x	x		0	0.00 %	1	0.02 %
16p11.2 del	x	x	x	6	0.03 %	3	0.06 %
16p11.2 dup	x	x	x	5	0.02 %	3	0.06 %
17p12 del (HNPP)			x	5	0.02 %	4	0.08 %
17p12 dup (CMT1A)			x	1	0.00 %	2	0.04 %
17q12 del			x	1	0.00 %	0	0.00 %
17q12 dup	x		x	2	0.01 %	2	0.04 %
22q11.2 dup	x	x	x	8	0.03 %	5	0.10 %
22q11.2 del	x	x	x	0	0.00 %	1	0.02 %
