# Supplementary material for: Polygenic burden has broader impact on health, cognition, and socioeconomic outcomes than most rare and high-risk copy number variants
Source: Mol Psychiatry. 2021 Feb 1;26(9):4884–95. doi: 10.1038/s41380-021-01026-z (PMC8589645; doi:10.1038/s41380-021-01026-z)
Supplement: Supplementary file 4 — Supplementary Table 3: DECIPHER disease-associated CNV regions and frequencies [file 41380_2021_1026_MOESM4_ESM.pdf]

DECIPHER CNVs in FINRISK and NFBC

Name	Chr	Start (hg19)	End (hg19)	Del/Dup	Size (kb)	n(FINRISK)	f(FINRISK)	n(NFBC)	f(NFBC)	Literature estimate*	Source	DOI
1p36 microdeletion syndrome	1	10001	12840259	DEL	12,830	0	0.0 %	0	0.0 %	*0.020 %*	Heilstedt et al 2003	10.1034/j.1399-0004.2003.00126.x
1q21.1 susceptibility locus for Thrombocytopenia-Absent Radius (TAR) syndrome	1	145386506	145748067	DEL	362	0	0.0 %	1	0.020 %	*0.018 %*	Kendall et al 2019	10.1192/bjp.2018.301
1q21.1 recurrent microdeletion (susceptibility locus for neurodevelopmental disorders)	1	146533376	147883376	DEL/DUP	1,350	12	0.052 %	4	0.082 %	*0.069 %*	Kendall et al 2019	10.1192/bjp.2018.301
2p21 Microdeletion Syndrome	2	44410451	44589584	DEL	179	0	0.0 %	0	0.0 %	*Not reported outside the Middle East*	Parvari et al 2001	10.1086/323624
2p15-16.1 microdeletion syndrome	2	59285696	61819815	DEL	2,534	0	0.0 %	0	0.0 %	*33 cases*	Bagheri et al 2016	10.1172/jci.insight.85461
2q33.1 deletion syndrome	2	196925121	205206939	DEL	8,282	0	0.0 %	0	0.0 %	*13 cases*	van Buggenhout et al 2005	10.1016/j.ejmg.2005.05.005
2q37 monosomy	2	239969863	240322643	DEL	353	0	0.0 %	0	0.0 %	*150 cases*	ORPHANET	N/A
3q29 microdeletion syndrome	3	195726835	197344663	DEL	1,618	0	0.0 %	0	0.0 %	*0.0033 %*	Kendall et al 2017	10.1016/j.biopsych.2016.08.014
Wolf-Hirschhorn Syndrome	4	1569197	2110236	DEL	541	0	0.0 %	0	0.0 %	*0.0010 %*	Shannon, Maltby, Rigby nad Quarrell 2001	10.1136/jmg.38.10.674
Cri du Chat Syndrome (5p deletion)	5	10001	12533304	DEL	12,523	0	0.0 %	0	0.0 %	*0.0022 %*	Niebuhr 1978	10.1007/BF00394291
Familial Adenomatous Polyposis	5	112043201	112181936	DEL	139	0	0.0 %	0	0.0 %	*0.00027 %*	ORPHANET/DECIPHER (Firth et al 2009)**	10.1016/j.ajhg.2009.03.010
Adult-onset autosomal dominant leukodystrophy (ADLD)	5	126112314	126172712	DEL	60	*SMALLER THAN DETECTION RESOLUTION*				*20 families in different ethnic groups*	ORPHANET	N/A
Sotos syndrome	5	175724636	177052116	DEL	1,327	0	0.0 %	0	0.0 %	*0.009 %*	ORPHANET	N/A
Williams-Beuren Syndrome (WBS)	7	72744455	74142672	DEL/DUP	1,398	0	0.0 %	0	0.0 %	*0.013 %*	Stromme et al 2002	10.1177/088307380201700406
Split hand/foot malformation 1 (SHFM1)	7	96318078	96339203	DEL	21	*SMALLER THAN DETECTION RESOLUTION*				*0.0090 %*	ORPHANET	N/A
8p23.1 deletion syndrome	8	8100055	11764629	DEL/DUP	3,665	0	0.0 %	0	0.0 %	*50 cases*	ORPHANET	N/A
8q21.11 Microdeletion Syndrome	8	77226464	77766239	DEL	540	0	0.0 %	0	0.0 %	*0.0090 %*	ORPHANET	N/A
9q subtelomeric deletion syndrome	9	140513443	140730578	DEL	217	0	0.0 %	0	0.0 %	*114 cases*	ORPHANET	N/A
WAGR 11p13 deletion syndrome	11	31806339	32457087	DEL	651	0	0.0 %	0	0.0 %	*0.00020 %*	DECIPHER (Firth et al 2009)	10.1016/j.ajhg.2009.03.010
Potocki-Shaffer syndrome	11	43994800	46052450	DEL	2,058	0	0.0 %	0	0.0 %	*23 cases*	ORPHANET	N/A
12p13.33 Microdeletion Syndrome	12	1080000	1346471	DEL	266	2	0.0087 %	1	0.020 %	*0.0001 %*	ORPHANET	N/A
12q14 microdeletion syndrome	12	65071919	68645525	DEL	3,574	0	0.0 %	0	0.0 %	*0.0001 %*	ORPHANET	N/A
Prader-Willi/Angelman syndrome (Type 1)	15	22749354	28438266	DEL/DUP	5,689	0	0.0 %	0	0.0 %	*0.0059 %*	Kendall et al 2017	10.1016/j.biopsych.2016.08.014
Prader-Willi/Angelman syndrome (Type 2)	15	23619912	28438266	DEL/DUP	4,818	1	0.0043 %	0	0.0 %	*0.0059 %*	Kendall et al 2017	10.1016/j.biopsych.2016.08.014
15q13.3 microdeletion syndrome	15	30910306	32445407	DEL	1,535	4	0.017 %	1	0.020 %	*0.0073 %*	Kendall et al 2017	10.1016/j.biopsych.2016.08.014
15q24 recurrent microdeletion syndrome	15	74412643	75972911	DEL	1,560	0	0.0 %	1	0.020 %	*0.00066 %*	Kendall et al 2017	10.1016/j.biopsych.2016.08.014
15q26 overgrowth syndrome	15	99357970	102521392	DEL	3,163	0	0.0 %	0	0.0 %	*14 cases*	Tatton-Brown et al 2009	10.1002/ajmg.a.32534
ATR-16 syndrome	16	60001	834372	DEL	774	0	0.0 %	0	0.0 %	*0.0001 %*	ORPHANET	N/A
Rubinstein-Taybi Syndrome	16	3775055	3930121	DEL	155	0	0.0 %	0	0.0 %	*0.0010 %*	ORPHANET	N/A
16p13.11 recurrent microdeletion (neurocognitive disorder susceptibility locus)	16	14986684	16486684	DEL/DUP	1,500	9	0.039 %	8	0.16 %	*0.034 %*	Kendall et al 2017	10.1016/j.biopsych.2016.08.014
16p11.2-p12.2 microduplication syndrome	16	21475060	29284077	DUP	7,809	0	0.0 %	0	0.0 %	*12 cases*	Barber et al 2013	10.1038/ejhg.2012.144
16p11.2-p12.2 microdeletion syndrome	16	21512062	30199854	DEL	8,688	0	0.0 %	0	0.0 %	*4 cases*	Ballif et al 2007	10.1038/ng2107
Recurrent 16p12.1 microdeletion (neurodevelopmental susceptibility locus)	16	21946524	22467284	DEL	521	17	0.074 %	6	0.12 %	*0.054 %*	Girirajan et al 2010	10.1038/ng.534
16p11.2 microduplication syndrome	16	29606852	30199855	DUP	593	4	0.017 %	3	0.061 %	*0.030 %*	Kendall et al 2017	10.1016/j.biopsych.2016.08.014
Miller-Dieker syndrome (MDS)	17	1	2588909	DEL	2,589	0	0.0 %	0	0.0 %	*0.0010 %*	ORPHANET	N/A
Charcot-Marie-Tooth syndrome type 1A (CMT1A)	17	14097915	15470903	DUP	1,373	1	0.0043 %	2	0.041 %	*0.030 %*	Kendall et al 2019	10.1192/bjp.2018.301
Hereditary Liability to Pressure Palsies (HNPP)	17	14097915	15470903	DEL	1,373	5	0.022 %	4	0.082 %	*0.056 %*	Kendall et al 2019	10.1192/bjp.2018.301
Smith-Magenis Syndrome	17	16773072	20222149	DEL	3,449	0	0.0 %	0	0.0 %	*0.0040 %*	Juyal et al 1996	N/A
Potocki-Lupski syndrome (17p11.2 duplication syndrome)	17	16773072	20222149	DUP	3,449	0	0.0 %	0	0.0 %	*0.00066 %*	Kendall et al 2017	10.1016/j.biopsych.2016.08.014
NF1-microdeletion syndrome	17	29107097	30263321	DEL	1,156	0	0.0 %	0	0.0 %	*0.0026 %*	Kendall et al 2017	10.1016/j.biopsych.2016.08.014
RCAD (renal cysts and diabetes)	17	34815072	36215917	DEL	1,401	1	0.0043 %	0	0.0 %	*0.00066 %*	Kendall et al 2017	10.1016/j.biopsych.2016.08.014
17q21.31 recurrent microdeletion syndrome (Koolen de Vries syndrome)	17	43705166	44294406	DEL	589	0	0.0 %	0	0.0 %	*0.0053 %*	Kendall et al 2017	10.1016/j.biopsych.2016.08.014
Early-onset Alzheimer disease with cerebral amyloid angiopathy	21	27252860	27543446	DUP	291	1	0.0043 %	0	0.0 %	*0.009 %*	ORPHANET	N/A
Cat-Eye Syndrome (Type I)	22	1	16971860	DUP	16,972	0	0.0 %	0	0.0 %	*0.0020 %*	ORPHANET	N/A
22q11 deletion syndrome (Velocardiofacial / DiGeorge syndrome)	22	19009792	21452445	DEL	2,443	0	0.0 %	1	0.020 %	*0.0033 %*	Kendall et al 2017	10.1016/j.biopsych.2016.08.014
22q11 duplication syndrome	22	19009792	21452445	DUP	2,443	7	0.030 %	5	0.10 %	*0.066 %*	Kendall et al 2019	10.1192/bjp.2018.301
22q11.2 distal deletion syndrome	22	21917117	23722445	DEL	1,805	0	0.0 %	0	0.0 %	*0.0020 %*	Kendall et al 2017	10.1016/j.biopsych.2016.08.014
22q13 deletion syndrome (Phelan-Mcdermid syndrome)	22	51045516	51187844	DEL	142	0	0.0 %	0	0.0 %	*Unknown*	ORPHANET	N/A

*Note: the frequency estimates are derived from very different samples. E.g. Kendall et al uses the UK Biobank, whereas most others are case-control studies

**While the estimated frequency of FAP in Europe is an estimated 1/11300-1/37600, the majority (70 %) of these carry a known (non-CNV mutation). CNV frequency for this syndrome is rarely solely reported, and this estimate is therefore non-representative
