# Supplementary material for: Polygenic burden has broader impact on health, cognition, and socioeconomic outcomes than most rare and high-risk copy number variants
Source: Mol Psychiatry. 2021 Feb 1;26(9):4884–95. doi: 10.1038/s41380-021-01026-z (PMC8589645; doi:10.1038/s41380-021-01026-z)
Supplement: Supplementary file 5 — Supplementary Table 4: Charlson Comorbidity Phenotypes [file 41380_2021_1026_MOESM5_ESM.pdf]

Endpoint	ICD-10	ICD-9
Myocardial infarction	I21-I23	410-411
Congestive heart failure	I50, I11.0, I13.0, I13.2	398, 402, 428
Peripheral vascular disease	I70-I74, I77	440-443
Cerebrovascular disease	I60-I69, G45-G46	430-433, 435
Dementia	F00-F03, F05.1, G30	290,291.2A, 291.2C, 294.1A
Chronic pulmonary disease	J40-J47, J60-J67, J68.4, J70.1, J70.3, J84.1, J92.0, J96.1, J98.2, J98.3	491-495, 515-518
Connective tissue disease	M05-M06, M08-M09, M30-M36, D86	710, 714, 725
Ulcer disease	K22.1, K25-K28	531-534
Mild liver disease	B18, K70.0-K70.3, K70.9, K71, K73, K74, K76.0	571, 573
Diabetes w/o end organ disease	E10.0, E10.1, E10.9, E11.0, E11.1, E11.9	
Diabetes with end organ danage	E10.2-E10.8, E11.2-E11.8	250
Hemiplegia	G81-G82	342, 434, 436, 437
Moderate to severe renal disease	I12, I13, N00-N05, N07, N11, N14, N17-N19, Q61	403, 404, 580-587
Non-metastatic solid tumour	C00-C69, C70-C75	140-189, 190-195
Leukaemia	C91-C95	204-208
Lymphoma	C81-C85, C88, C90, C96	200, 202, 203
Moderate to severe liver disease	B15.0, B16.0, B16.2, B19.0, K70.4, K72, K76.6, I85	070, 570, 572
Metastatic cancer	C76-C79, C80	199
AIDS	B21-B24	

Phenotype selections used in compilation of Charlson comorbidity index (CCI). CCI was calculated for each listed phenotype, corresponding to the appearance of a comorbidity belonging to that category during follow-up time. Total individual CCI was determined as the sum of all phenotype CCIs without discrimination or ranking.
