# Supplementary material for: Polygenic burden has broader impact on health, cognition, and socioeconomic outcomes than most rare and high-risk copy number variants
Source: Mol Psychiatry. 2021 Feb 1;26(9):4884–95. doi: 10.1038/s41380-021-01026-z (PMC8589645; doi:10.1038/s41380-021-01026-z)
Supplement: Supplementary file 7 — Supplementary Figure 1: Correlation between neuropsychiatric disorders in FINRISK [file 41380_2021_1026_MOESM7_ESM.pdf]

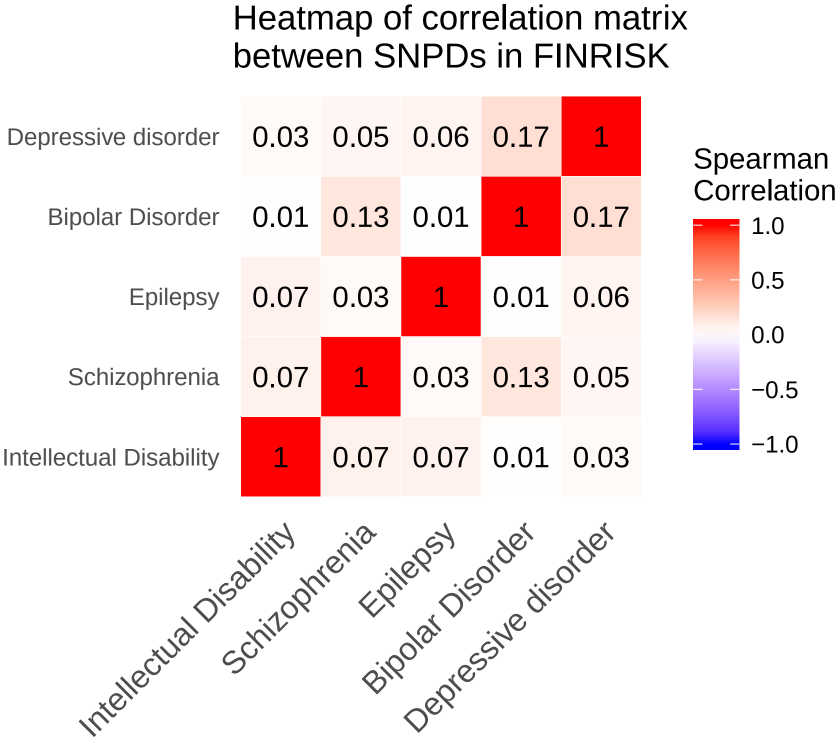
Heatmap of correlation matrix
between SNPDs in FINRISK
Depressive disorder
Bipolar Disorder
Epilepsy
Schizophrenia
Intellectual Disability
0.03 0.05 0.06 0.17 1
0.01 0.13 0.01 1 0.17
0.07 0.03 1 0.01 0.06
0.07 1 0.03 0.13 0.05
1 0.07 0.07 0.01 0.03
Intellectual Disability
Schizophrenia
Epilepsy
Bipolar Disorder
Depressive disorder
Spearman
Correlation
1.0
0.5
0.0
−0.5
−1.0
