# Supplementary material for: Polygenic burden has broader impact on health, cognition, and socioeconomic outcomes than most rare and high-risk copy number variants
Source: Mol Psychiatry. 2021 Feb 1;26(9):4884–95. doi: 10.1038/s41380-021-01026-z (PMC8589645; doi:10.1038/s41380-021-01026-z)
Supplement: Supplementary file 8 — Supplementary Figure 2: Meta-analysis of SNPD association with CNV subgroups [file 41380_2021_1026_MOESM8_ESM.pdf]

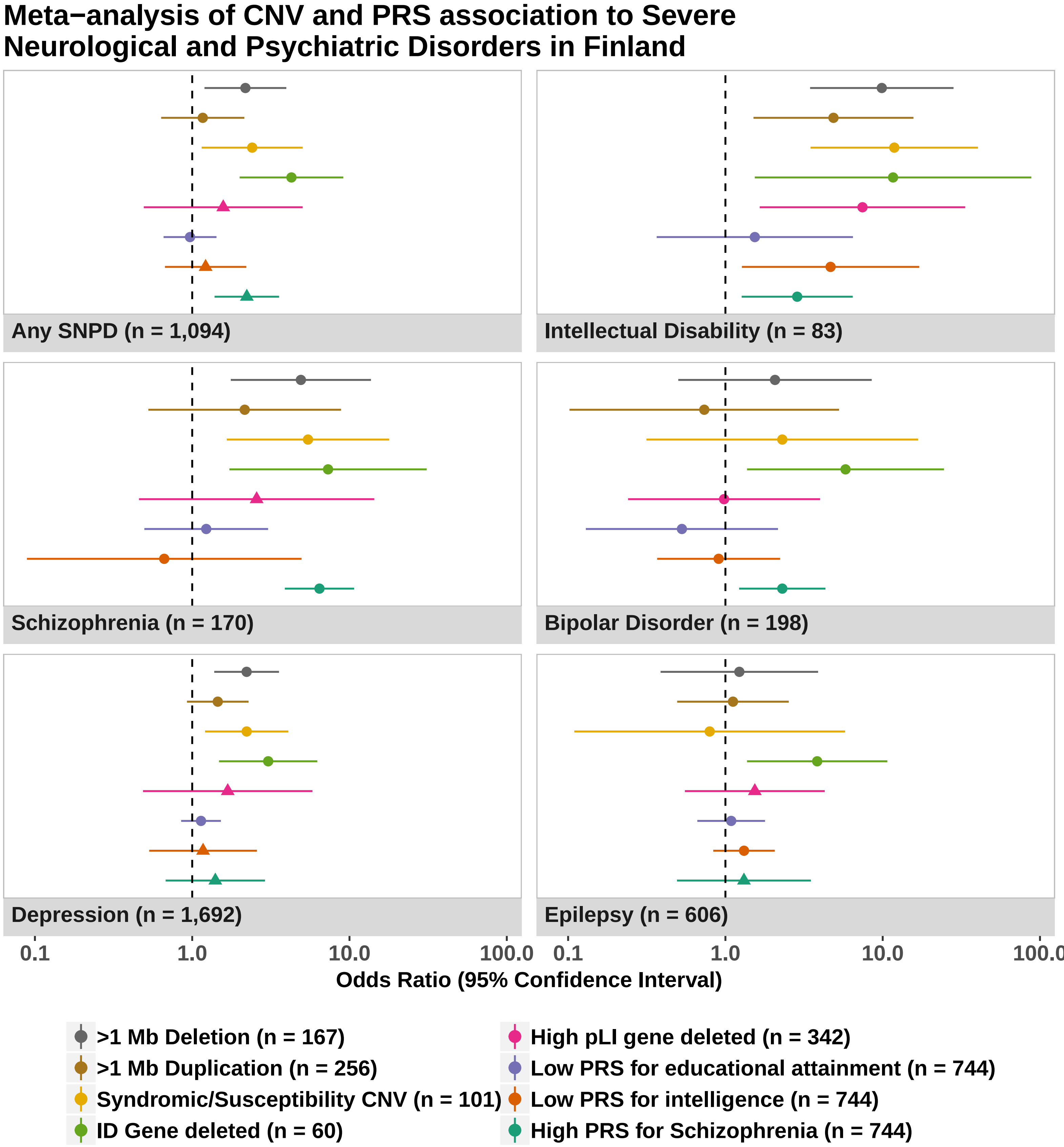
Meta−analysis of CNV and PRS association to Severe Neurological and Psychiatric Disorders in Finland
Any SNPD (n = 1,094)
Intellectual Disability (n = 83)
Schizophrenia (n = 170)
Bipolar Disorder (n = 198)
Depression (n = 1,692)
Epilepsy (n = 606)
0.1
1.0
10.0
100.0
Odds Ratio (95% Confidence Interval)
>1 Mb Deletion (n = 167)
>1 Mb Duplication (n = 256)
Syndromic/Susceptibility CNV (n = 101)
ID Gene deleted (n = 60)
High pLI gene deleted (n = 342)
Low PRS for educational attainment (n = 744)
Low PRS for intelligence (n = 744)
High PRS for Schizophrenia (n = 744)
