# Supplementary material for: Polygenic burden has broader impact on health, cognition, and socioeconomic outcomes than most rare and high-risk copy number variants
Source: Mol Psychiatry. 2021 Feb 1;26(9):4884–95. doi: 10.1038/s41380-021-01026-z (PMC8589645; doi:10.1038/s41380-021-01026-z)

CNV and PRS association to severe neurological and psychiatric disorders in FINRISK (n = 23,053)

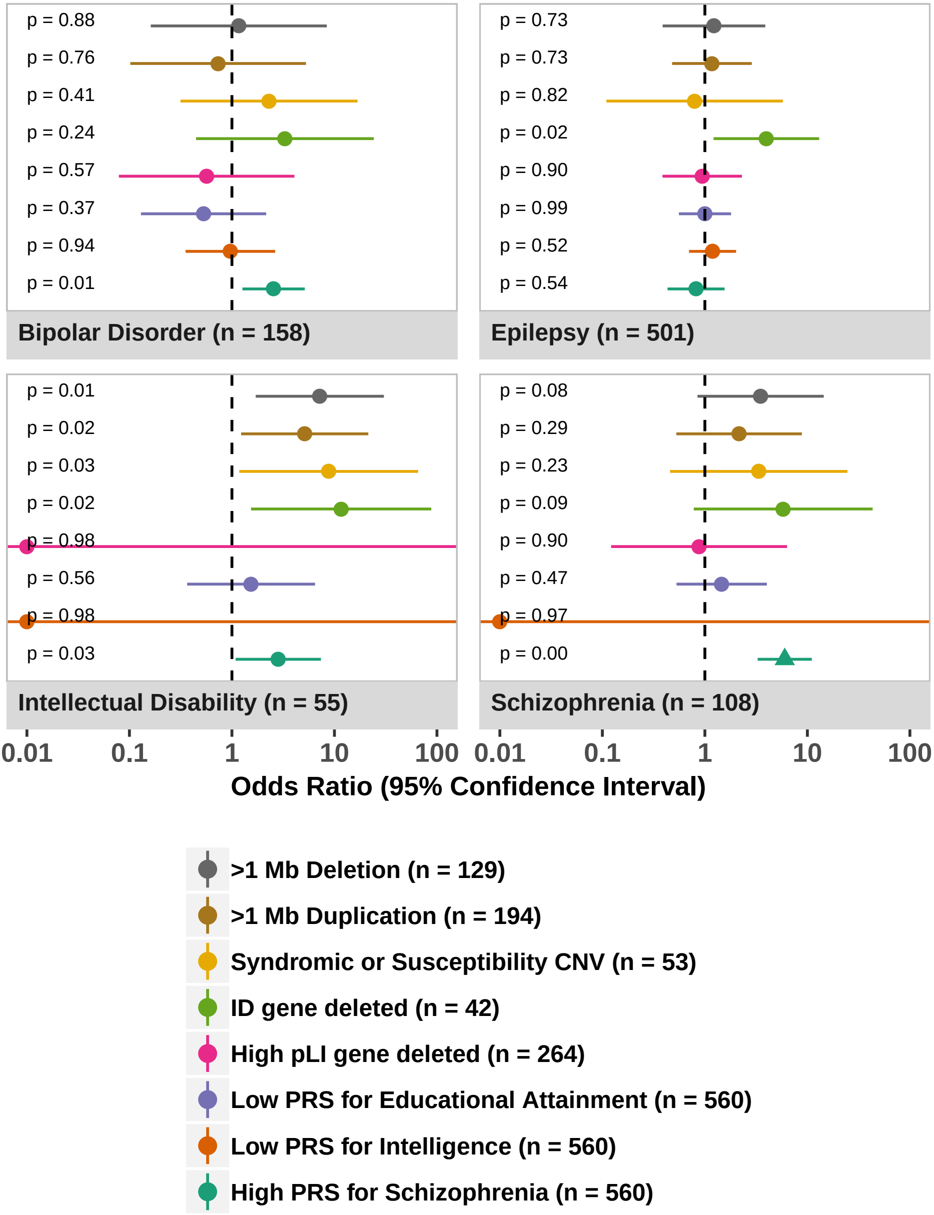

Supplement: Supplementary file 9 — Supplementary Figure 3: SNPD association with CNV subgroups in FINRISK [file 41380_2021_1026_MOESM9_ESM.pdf]
