# Supplementary figures and images for: Polygenic burden has broader impact on health, cognition, and socioeconomic outcomes than most rare and high-risk copy number variants
Source: Mol Psychiatry. 2021 Feb 1;26(9):4884–95. doi: 10.1038/s41380-021-01026-z (PMC8589645; doi:10.1038/s41380-021-01026-z)

Heatmap of correlation matrix between socioeconomic endpoints in FINRISK

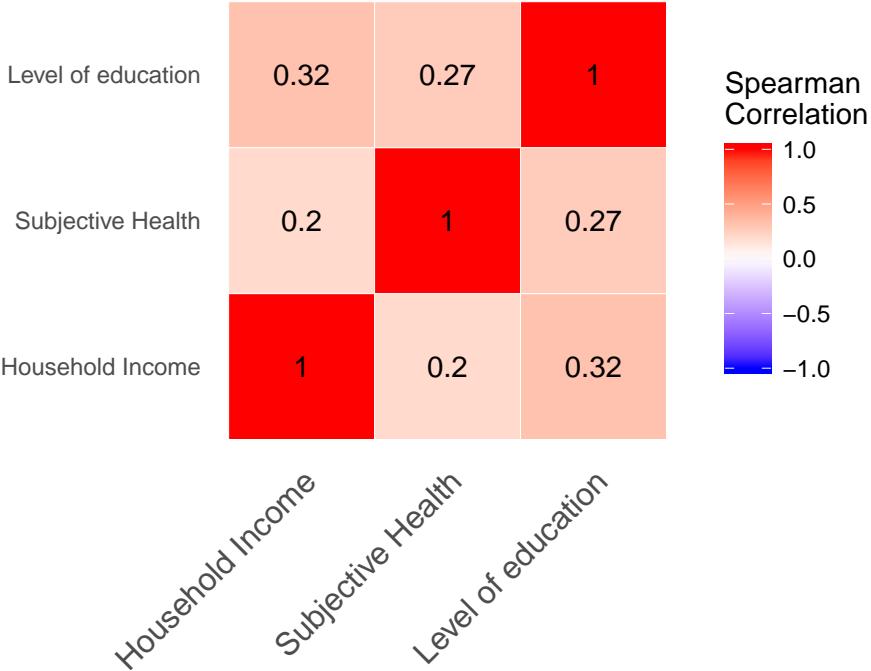

Supplement: Supplementary file 11 — Supplementary Figure 5: Spearman’s correlation between categorical socioeconomic endpoints in FINRISK [file 41380_2021_1026_MOESM11_ESM.pdf]

# CNV and PRS association with

Household Income

Income (education covariate)

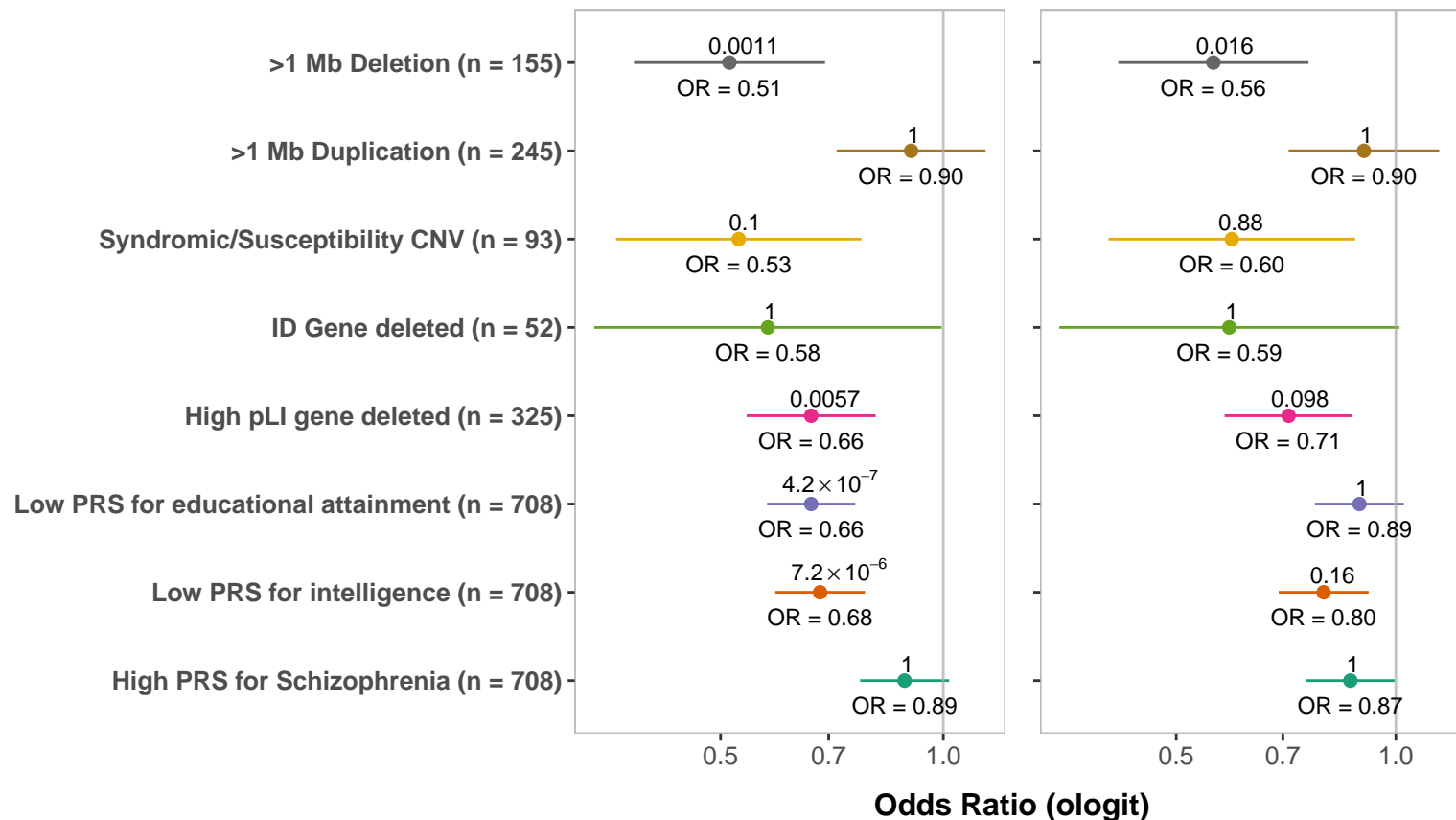

Supplement: Supplementary file 12 — Supplementary Figure 6: Meta-analysis of level of household income in CNV subgroups [file 41380_2021_1026_MOESM12_ESM.pdf]

# CNV and PRS association with Subjective Health in NFBC66

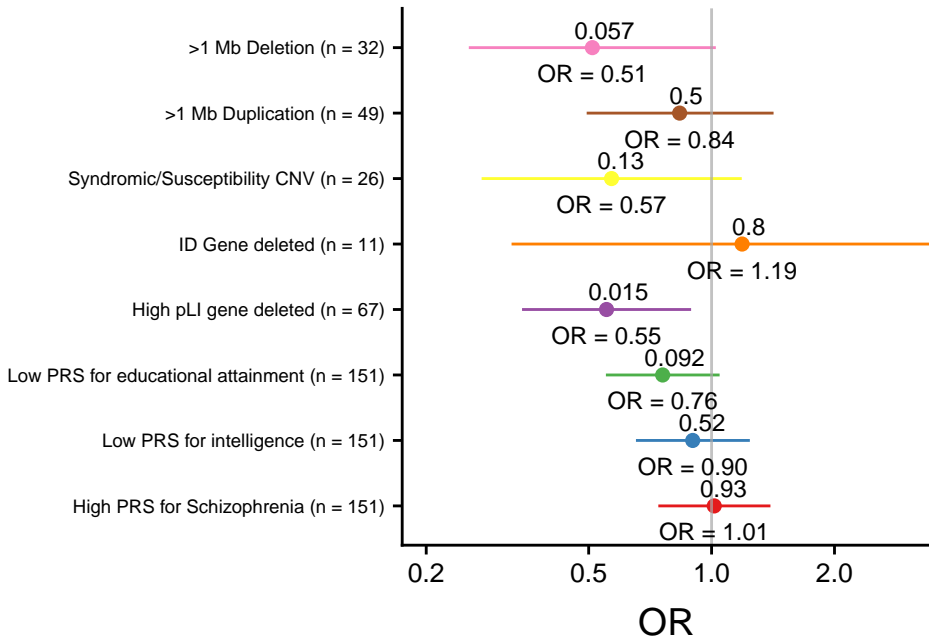

Supplement: Supplementary file 13 — Supplementary Figure 7: Subjective health in different CNV subgroups in NFBC [file 41380_2021_1026_MOESM13_ESM.pdf]

# CNV and PRS association with Subjective Health

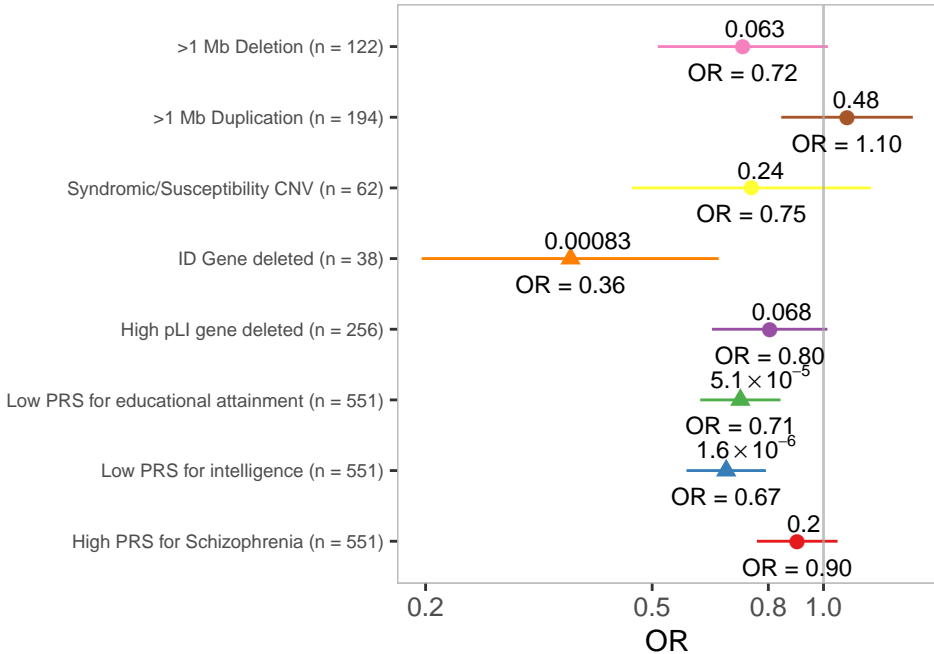

Supplement: Supplementary file 14 — Supplementary Figure 8: Subjective health in different CNV subgroups in FINRISK [file 41380_2021_1026_MOESM14_ESM.pdf]

# Charlson Comorbidity Index

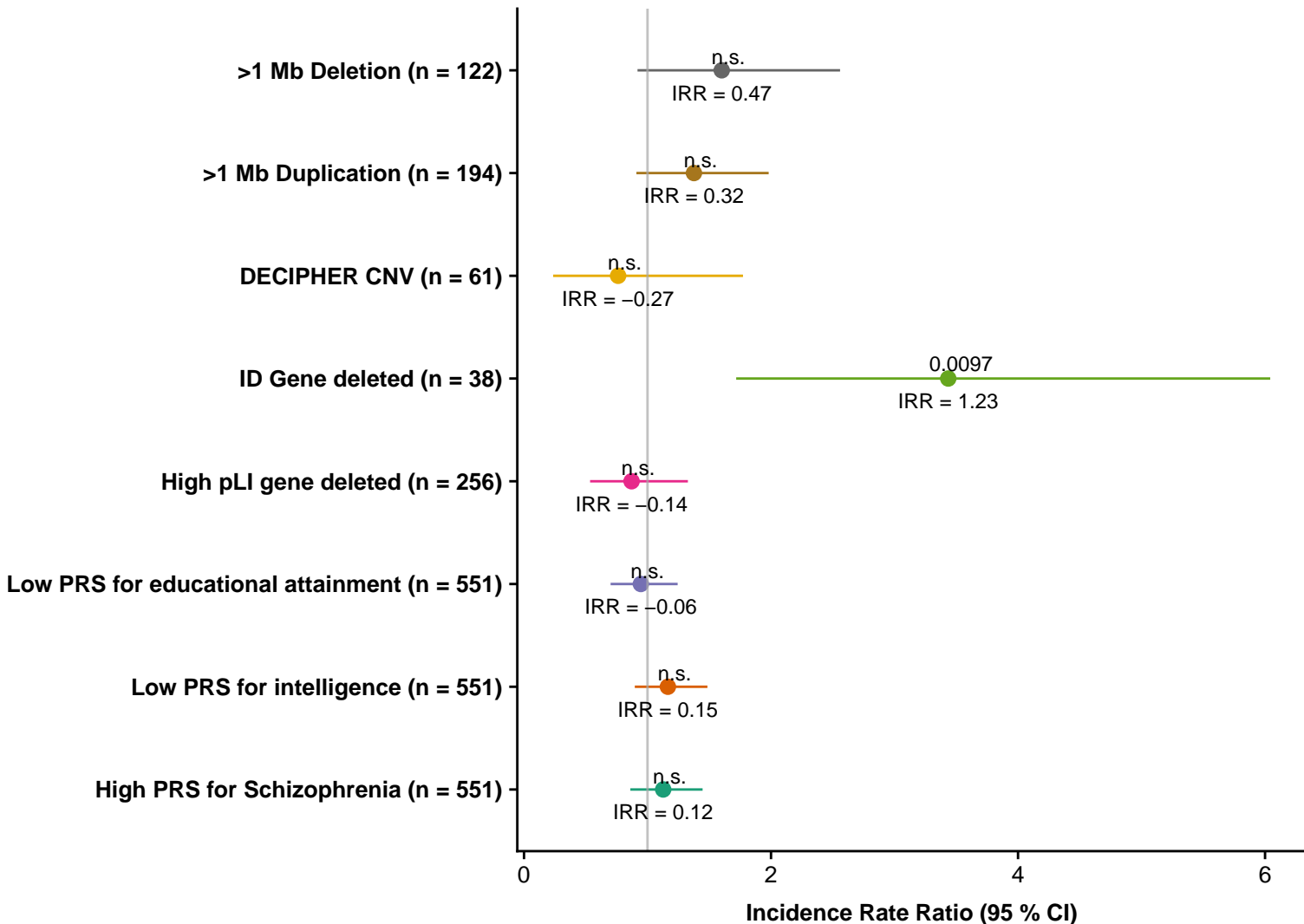

Supplement: Supplementary file 15 — Supplementary Figure 9: Charlson Comorbidity Index in different CNV subgroups in FINRISK [file 41380_2021_1026_MOESM15_ESM.pdf]

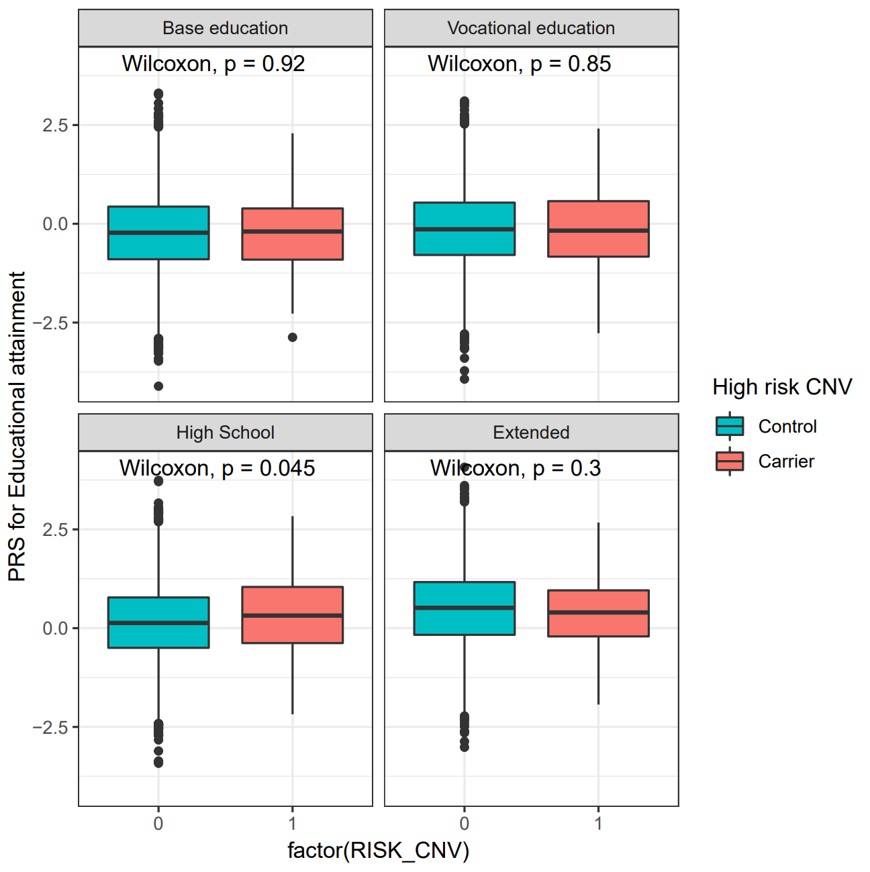

Supplement: Supplementary file 17 — Supplementary Figure 11: PRS_EA distribution in FINRISK CNV carriers vs non-carriers not affected by SNPD, by level of education [file 41380_2021_1026_MOESM17_ESM.jpg]

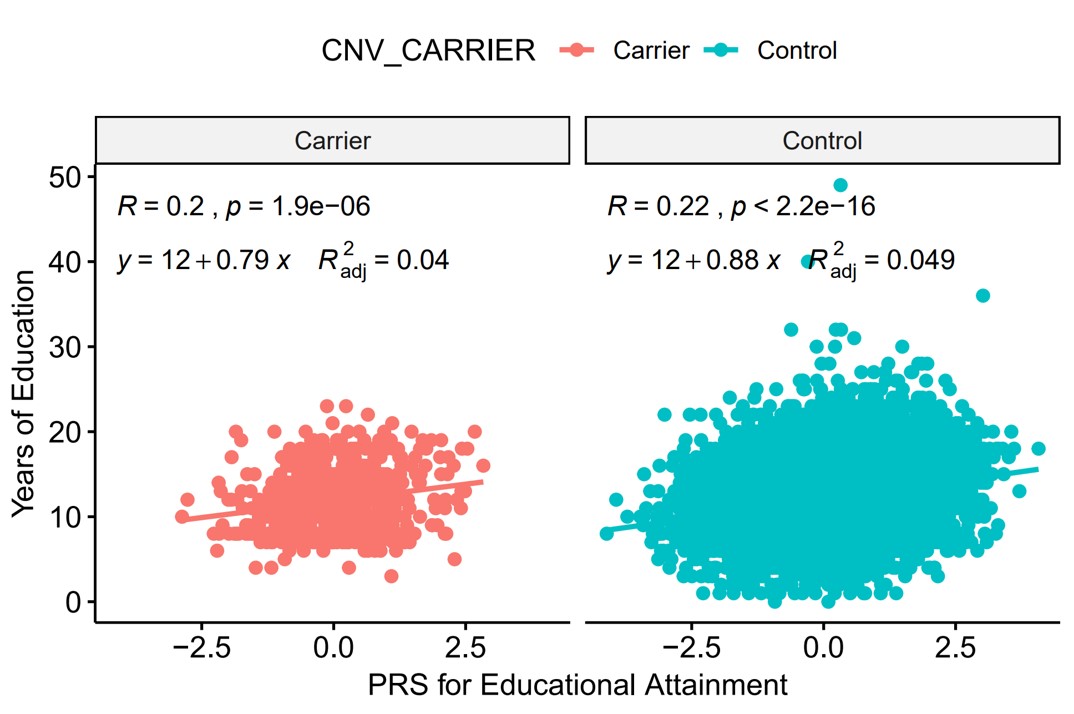

Supplement: Supplementary file 18 — Supplementary Figure 12: PRS_EA distribution in FINRISK CNV carriers vs non-carriers not affected by SNPD, by years of education [file 41380_2021_1026_MOESM18_ESM.jpg]
