# Supplementary material for: Polygenic burden has broader impact on health, cognition, and socioeconomic outcomes than most rare and high-risk copy number variants
Source: Mol Psychiatry. 2021 Feb 1;26(9):4884–95. doi: 10.1038/s41380-021-01026-z (PMC8589645; doi:10.1038/s41380-021-01026-z)
Supplement: Supplementary file 16 — Supplementary Figure 10: Meta-analysis of impact of high-risk CNVs and PRS outlier status on socioeconomic grouping [file 41380_2021_1026_MOESM16_ESM.pdf]

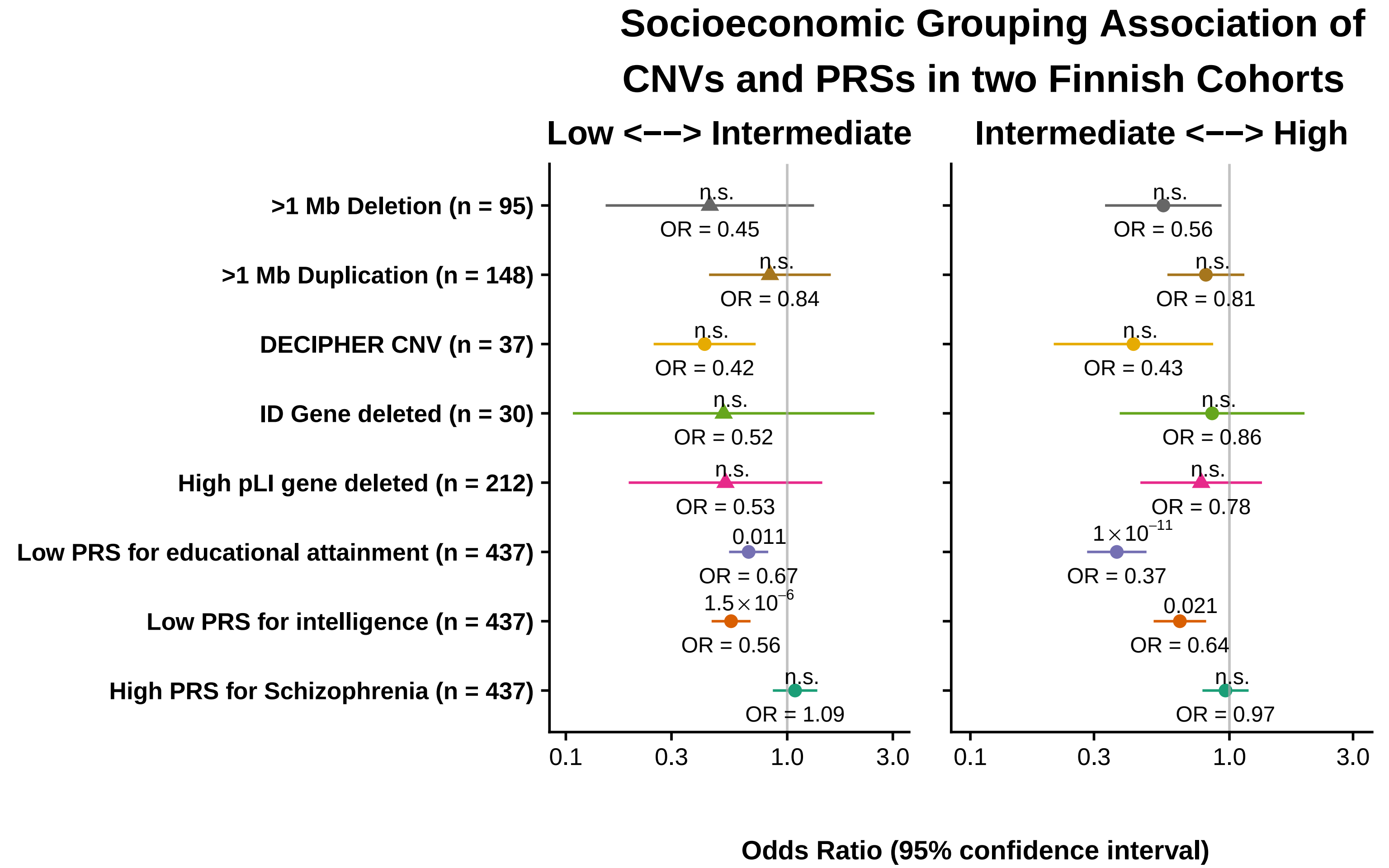

Socioeconomic Grouping Association of CNVs and PRSs in two Finnish Cohorts
Low <--> Intermediate
Intermediate <--> High
>1 Mb Deletion (n = 95)
>1 Mb Duplication (n = 148)
DECIPHER CNV (n = 37)
ID Gene deleted (n = 30)
High pLI gene deleted (n = 212)
Low PRS for educational attainment (n = 437)
Low PRS for intelligence (n = 437)
High PRS for Schizophrenia (n = 437)
n.s.
OR = 0.45
OR = 0.56
OR = 0.84
OR = 0.81
OR = 0.42
OR = 0.43
OR = 0.52
OR = 0.86
OR = 0.53
OR = 0.78
0.011
OR = 0.67
1 × 10⁻¹¹
OR = 0.37
1.5 × 10⁻⁶
OR = 0.56
0.021
OR = 0.64
OR = 1.09
OR = 0.97
0.1
0.3
1.0
3.0
Odds Ratio (95% confidence interval)
